# The 14-3-3 protein OsGF14f interacts with OsbZIP23 and enhances its activity to confer osmotic stress tolerance in rice

**DOI:** 10.1093/plcell/koad211

**Published:** 2023-07-28

**Authors:** Yamei Ma, Ziying Wu, Jingfang Dong, Shaohong Zhang, Junliang Zhao, Tifeng Yang, Wu Yang, Lian Zhou, Jian Wang, Jiansong Chen, Qing Liu, Bin Liu

**Affiliations:** Rice Research Institute, Guangdong Academy of Agricultural Sciences, Key Laboratory of Genetics and Breeding of High Quality Rice in Southern China (Co-construction by Ministry and Province), Ministry of Agriculture and Rural Affairs, Guangdong Key Laboratory of New Technology in Rice Breeding, Guangdong Rice Engineering Laboratory, Guangzhou 510640, Guangdong,China; Rice Research Institute, Guangdong Academy of Agricultural Sciences, Key Laboratory of Genetics and Breeding of High Quality Rice in Southern China (Co-construction by Ministry and Province), Ministry of Agriculture and Rural Affairs, Guangdong Key Laboratory of New Technology in Rice Breeding, Guangdong Rice Engineering Laboratory, Guangzhou 510640, Guangdong,China; Rice Research Institute, Guangdong Academy of Agricultural Sciences, Key Laboratory of Genetics and Breeding of High Quality Rice in Southern China (Co-construction by Ministry and Province), Ministry of Agriculture and Rural Affairs, Guangdong Key Laboratory of New Technology in Rice Breeding, Guangdong Rice Engineering Laboratory, Guangzhou 510640, Guangdong,China; Rice Research Institute, Guangdong Academy of Agricultural Sciences, Key Laboratory of Genetics and Breeding of High Quality Rice in Southern China (Co-construction by Ministry and Province), Ministry of Agriculture and Rural Affairs, Guangdong Key Laboratory of New Technology in Rice Breeding, Guangdong Rice Engineering Laboratory, Guangzhou 510640, Guangdong,China; Rice Research Institute, Guangdong Academy of Agricultural Sciences, Key Laboratory of Genetics and Breeding of High Quality Rice in Southern China (Co-construction by Ministry and Province), Ministry of Agriculture and Rural Affairs, Guangdong Key Laboratory of New Technology in Rice Breeding, Guangdong Rice Engineering Laboratory, Guangzhou 510640, Guangdong,China; Rice Research Institute, Guangdong Academy of Agricultural Sciences, Key Laboratory of Genetics and Breeding of High Quality Rice in Southern China (Co-construction by Ministry and Province), Ministry of Agriculture and Rural Affairs, Guangdong Key Laboratory of New Technology in Rice Breeding, Guangdong Rice Engineering Laboratory, Guangzhou 510640, Guangdong,China; Rice Research Institute, Guangdong Academy of Agricultural Sciences, Key Laboratory of Genetics and Breeding of High Quality Rice in Southern China (Co-construction by Ministry and Province), Ministry of Agriculture and Rural Affairs, Guangdong Key Laboratory of New Technology in Rice Breeding, Guangdong Rice Engineering Laboratory, Guangzhou 510640, Guangdong,China; Rice Research Institute, Guangdong Academy of Agricultural Sciences, Key Laboratory of Genetics and Breeding of High Quality Rice in Southern China (Co-construction by Ministry and Province), Ministry of Agriculture and Rural Affairs, Guangdong Key Laboratory of New Technology in Rice Breeding, Guangdong Rice Engineering Laboratory, Guangzhou 510640, Guangdong,China; Rice Research Institute, Guangdong Academy of Agricultural Sciences, Key Laboratory of Genetics and Breeding of High Quality Rice in Southern China (Co-construction by Ministry and Province), Ministry of Agriculture and Rural Affairs, Guangdong Key Laboratory of New Technology in Rice Breeding, Guangdong Rice Engineering Laboratory, Guangzhou 510640, Guangdong,China; Rice Research Institute, Guangdong Academy of Agricultural Sciences, Key Laboratory of Genetics and Breeding of High Quality Rice in Southern China (Co-construction by Ministry and Province), Ministry of Agriculture and Rural Affairs, Guangdong Key Laboratory of New Technology in Rice Breeding, Guangdong Rice Engineering Laboratory, Guangzhou 510640, Guangdong,China; Rice Research Institute, Guangdong Academy of Agricultural Sciences, Key Laboratory of Genetics and Breeding of High Quality Rice in Southern China (Co-construction by Ministry and Province), Ministry of Agriculture and Rural Affairs, Guangdong Key Laboratory of New Technology in Rice Breeding, Guangdong Rice Engineering Laboratory, Guangzhou 510640, Guangdong,China; Rice Research Institute, Guangdong Academy of Agricultural Sciences, Key Laboratory of Genetics and Breeding of High Quality Rice in Southern China (Co-construction by Ministry and Province), Ministry of Agriculture and Rural Affairs, Guangdong Key Laboratory of New Technology in Rice Breeding, Guangdong Rice Engineering Laboratory, Guangzhou 510640, Guangdong,China

## Abstract

Drought, which can induce osmotic stress, is the leading environmental constraint on crop productivity. Plants in both agricultural and natural settings have developed various mechanisms to cope with drought stress. The identification of genes associated with drought stress tolerance and understanding the underlying regulatory mechanisms are prerequisites for developing molecular manipulation strategies to address this issue. Here, we reported that the G-BOX FACTOR 14-3-3f (14-3-3 protein OsGF14f) positively modulates osmotic stress tolerance in rice (*Oryza sativa*). *OsGF14f* transgenic lines had no obvious change in crucial agronomic traits including yield and plant height. *OsGF14f* is transcriptionally induced by PEG treatment, and in rice, overexpression or knockout of this gene leads to enhanced or weakened osmotic stress tolerance, respectively. Furthermore, OsGF14f positively regulates abscisic acid (ABA) responses by interacting with the core ABA-responsive transcription factor BASIC LEUCINE ZIPPER 23 (OsbZIP23) to enhance its transcriptional regulation activity toward downstream target genes. Further genetic analysis showed that OsGF14f is required for the full function of OsbZIP23 in rice osmotic response, and OsGF14f-mediated osmotic stress tolerance partially depends on OsbZIP23. Interestingly, *OsGF14f* is a direct target gene of OsbZIP23. Taken together, our findings reveal a genetic and molecular framework by which the OsGF14f–OsbZIP23 complex modulates rice osmotic response, providing targets for developing drought-tolerant crops.

## Teaser

OsGF14f interacts with and enhances the transcriptional regulation activity of OsbZIP23 to positively regulate osmotic stress tolerance in rice.

## Introduction

The global demand for crop production is expected to roughly double by 2050 due to our growing population, diet shifts, and increasing biofuel use (Ray et al. 2012, 2013). However, the expected crop production growth rate falls short of the increasing demand (Ray et al. 2012, 2013). The achievement of higher and more consistent crop production relies on our ability to overcome the various environmental stresses that seriously hamper crop yield and quality. One of the most critical environmental stresses is drought, which contributes to over 40% of crop losses worldwide. This proportion is continuously increasing due to climate change (Fàbregas et al. 2018). Rice (*Oryza sativa*) is one of the most important food crops in the world, making it a critical target for drought tolerance improvement. It is also a semiaquatic plant, meaning that it requires much more intensive irrigation and a larger water supply than other cereal crops such as maize (*Zea mays*) and wheat (*Triticum aestivum*). Therefore, the improvement of drought tolerance is an important target in rice breeding.

Drought causes osmotic stress to cells, and the osmotic stress leads to reduced water uptake and dehydration (Nakashima and Yamaguchi-Shinozaki 2013). The tolerance of plants to drought-induced osmotic stress is derived from a series of complex traits controlled by multiple genes. In the past, numerous quantitative trait loci (QTLs) associated with drought tolerance have been identified and used in the development of drought-tolerant rice varieties. However, in previous studies, increased drought tolerance in transgenic plants has generally come at the cost of reduced growth (Nakashima et al. 2007; Shao et al. 2008; Tardieu 2012; Weng et al. 2014). Hence, the discovery of genes balancing rice growth and stress tolerance will hold promise for developing rice varieties with both drought tolerance and high yield.

14-3-3 proteins, a class of highly conserved, acidic, soluble proteins in all eukaryotes, are widely associated with plant growth and stress responses (Denison et al. 2011; Liu et al. 2016c). They generally work as molecular chaperones and can regulate the intracellular localization, protein stability, and protein activity of their binding partners to exert function (Denison et al. 2011; Liu et al. 2016c). The rice 14-3-3 family contains 8 members, some of which are reported to regulate rice growth and stress responses (Liu et al. 2016c). These rice proteins can interact with BRASSINAZOLE-RESISTANT 1 (OsBZR1), an important positive factor in brassinosteroid signaling, and reduce its nuclear localization, thereby regulating BR-regulated rice growth (Bai et al. 2007).

Recently, our studies have shown that 14-3-3s are involved in rice disease defense responses (Liu et al. 2016a, 2016b, 2016c; Yan et al. 2021; Ma, Yang et al. 2022). For example, G-BOX FACTOR 14-3-3e (OsGF14e) and G-BOX FACTOR 14-3-3b (OsGF14b) have been found to positively regulate panicle blast resistance in rice (Liu et al. 2016a, 2016b). Further studies showed that *OsGF14b* is a target gene of WRKY transcription factor 71 (OsWRKY71), which positively regulates rice resistance to panicle blast (Liu et al. 2016a). Proteomics and metabolomics analyses have revealed that OsGF14b can activate the gibberellin biosynthetic, auxin, and jasmonic acid signaling pathway during blast infection (Yan et al. 2021). Most recently, it has been discovered that OsGF14f positively regulates leaf blast and bacterial blight resistance in rice via the salicylic acid–dependent signaling pathway (Ma, Yang et al. 2022). Other labs have used a transgenic approach to experimentally confirm the role of G-BOX FACTOR 14-3-3c (OsGF14c) and OsGF14b in rice drought tolerance (Ho et al. 2013; Liu et al. 2019), while the details of the regulatory mechanisms involved in 14-3-3–mediated drought signal response in rice are still unclear.

Abscisic acid (ABA) is a well-known stress hormone that coordinates the complex networks of stress responses. Osmotic stress caused by drought stress rapidly triggers ABA accumulation in plants (Zhu 2002). Many proteins involved in the ABA-mediated stress signaling pathway have been identified (Lee and Luan 2012; Zhu 2016), among which several bZIP-type transcription factors have been found to function as key regulators, such as ABSCISIC ACID INSENSITIVE 5 (ABI5) in Arabidopsis (*Arabidopsis thaliana*) and OsbZIP23 in rice. The bZIP-type transcription factors regulate the expression of ABA-responsive genes by binding to the G-box or ABA-responsive elements (ABREs) in their promoters, triggering a series of physiological reactions to respond to environmental stimuli (Yoshida et al. 2010; Zong et al. 2016).

In rice, OsbZIP23 is acutely induced by drought, high salt, PEG, and ABA treatments (Xiang et al. 2008). Transgenic rice plants overexpressing *OsbZIP23* display increased sensitivity to ABA and improved drought and salinity tolerance, whereas the *osbzip23* mutant exhibits opposite phenotypes (Xiang et al. 2008). Genome-wide studies have revealed that OsbZIP23 can activate the expression of a variety of stress-responsive genes, for examples, *LATE EMBRYOGENESIS ABUNDANT PROTEIN 3-2* (*OsLEA3-2*) and *RESPONSIVE TO ABSCISIC ACID 16A* (*OsRab16A*) (Zong et al. 2016), thus positively modulating rice drought tolerance. Several studies have shown that the function of OsbZIP23 is tightly controlled by its interacting partners. For example, a SnRK2 protein kinase osmotic stress/ABA–activated protein kinase 2 (SAPK2) interacts with and phosphorylates OsbZIP23 to activate its transcriptional activity (Zong et al. 2016). SUMO protease OVERLY TOLERANT TO SALT 1 (OsOTS1) interacts with and de-SUMOylates OsbZIP23 to reduce its stability (Srivastava et al. 2017). Despite these findings, the exact mechanisms underlying OsbZIP23-mediated transcriptional regulation require further study.

In this study, we have shown that rice 14-3-3 protein OsGF14f is a positive regulator of osmotic stress tolerance. OsGF14f interacts with OsbZIP23 and enhances its transcriptional regulation function to modulate the expression of stress-responsive genes. Disruption of OsGF14f largely attenuates the osmotic stress-tolerant phenotype of the *OsbZIP23*-OE plants. In turn, OsGF14f-mediated osmotic stress tolerance is partially dependent on OsbZIP23. Interestingly, *OsGF14f* is a direct target gene of OsbZIP23, causing the feedbacked activation of OsbZIP23. Thus, these results unravel one of the molecular mechanisms regarding OsGF14f–OsbZIP23-mediated osmotic stress tolerance in rice.

## Results

### *OsGF14f* transcription is induced by osmotic stress

Our previous studies have revealed that 14-3-3 genes are involved in rice disease defense responses (Liu et al. 2016a, 2016b; Yan et al. 2021; Ma, Yang et al. 2022). In this study, we aim to further clarify the roles of these proteins during rice growth, development, and stress response. To begin, we analyzed the expression profiles of all 8 members of the rice 14-3-3 protein family based on the available transcriptomics databases (http://bar.utoronto.ca/; http://rice.uga.edu/). We found that all were ubiquitously expressed in the examined tissues, and *OsGF14f* showed a relatively higher expression than the others (Supplemental Fig. S1A). In addition, most 14-3-3 genes were actively responsive to at least 1 kind of abiotic stresses, such as drought, high salt, and cold (Supplemental Fig. S1B).

**Figure 1.**
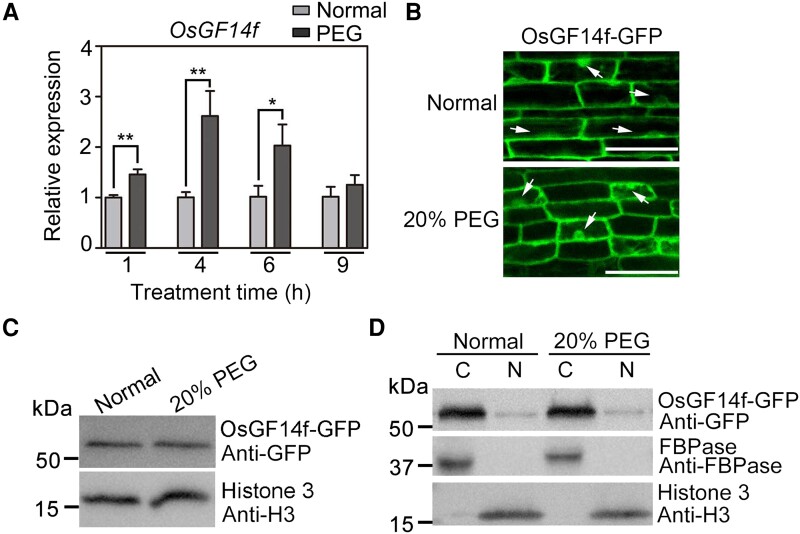
The response of *OsGF14f* transcripts and protein to osmotic stress treatment. **A)** RT-qPCR analysis showing *OsGF14f* gene expression in shoots following a 20% PEG6000 treatment. Two-week-old Nip seedlings were exposed to a 20% PEG6000 treatment and harvested at the indicated time points for total RNA extraction and gene expression analysis. Each sample contained 5 uniform seedlings. Data represents means ± Sd of 3 independent experiments. *P < 0.05 and **P < 0.01 (unpaired 2-tailed Student's *t* test). **B)** Confocal images of OsGF14f-GFP in root cells of *proUBQ10:OsGF14f-GFP* transgenic rice plants. Two-week-old seedlings were treated with 20% PEG6000 or a mock substance for 24 h, and then the GFP signal was captured using confocal microscopy. The arrow indicates the nucleus. Scale bars = 25 *μ*m. About 5 seedlings were observed with similar results. **C)** Immunoblot analysis of OsGF14f-GFP protein following a 20% PEG6000 treatment. Two-week-old *proUBQ10:OsGF14f-GFP* seedlings were treated with 20% PEG6000 or a mock substance for 24 h, and then total proteins were extracted and subjected to immunoblot analysis using anti-GFP and anti-H3 antibodies. **D)** The nucleo–cytoplasmic distribution of OsGF14f-GFP protein following a 20% PEG6000 treatment. Two-week-old *proUBQ10:OsGF14f-GFP* seedlings were treated with 20% PEG6000 or a mock substance for 24 h, and then the nuclear and cytosol fractionations were extracted for immunoblot analysis using an anti-GFP antibody. Histone 3 and FBPase were used as the nuclear and cytoplasm markers, respectively. C, cytoplasmic fraction; N, nuclear fraction.

Interestingly, *OsGF14f* was strongly induced by drought stress (Supplemental Fig. S1B), indicating that this gene may play an important role during rice drought response. To verify this, we used reverse transcription quantitative PCR (RT-qPCR) to monitor *OsGF14f* expression upon PEG-mimicked drought stress. As shown in Fig. 1A, the expression level of *OsGF14f* in shoots rapidly and significantly increased following 20% PEG6000 treatment, peaking at 4 h after treatment. In roots, *OsGF14f* showed increased expression at early stage (1 h) but decreased expression at late stages (4 to 9 h) following 20% PEG6000 treatment (Supplemental Fig. S2).

To determine whether osmotic stress treatment influences the subcellular localization and protein stability of OsGF14f, we generated transgenic rice plants expressing *OsGF14f-GFP* driven by a *UBQ10* promoter in Nipponbare (Nip) background. Confocal observation showed that OsGF14f-GFP protein was mainly distributed in the cytoplasm, with a small proportion in the nucleus under normal conditions (Fig. 1B). Moreover, the fluorescence intensity and localization pattern of OsGF14f-GFP were not noticeably altered after a 24-h PEG6000 treatment (Fig. 1B). These results were further confirmed by an immunoblotting assay (Fig. 1, C and D). As shown in Fig. 1C, the total amount of OsGF14f-GFP protein was not changed after 24-h PEG6000 treatment. Furthermore, a cell fractionation assay revealed that 24-h PEG6000 treatment did not affect the nucleolus–cytoplasmic distribution of OsGF14f-GFP (Fig. 1D). Collectively, these results indicate that the influence of osmotic stress on *OsGF14f* mainly occurs at the transcriptional level.

### OsGF14f positively regulates osmotic stress tolerance in rice

To dissect the detailed biological functions of *OsGF14f* in rice, we have previously generated transgenic rice plants, which overexpress *OsGF14f* (Ma, Yang et al. 2022). Three independent homozygous lines (OE-2, OE-3, and OE-5) were selected for phenotypic analyses. Under normal conditions, there were no significant phenotypic differences between the 3 OE lines and the wild-type Nip plants, including the plant height, tillering number, and grain yield per plant (Supplemental Fig. S3). Therefore, these 3 lines were used for further study of osmotic stress tolerance in rice.

**Figure 2.**
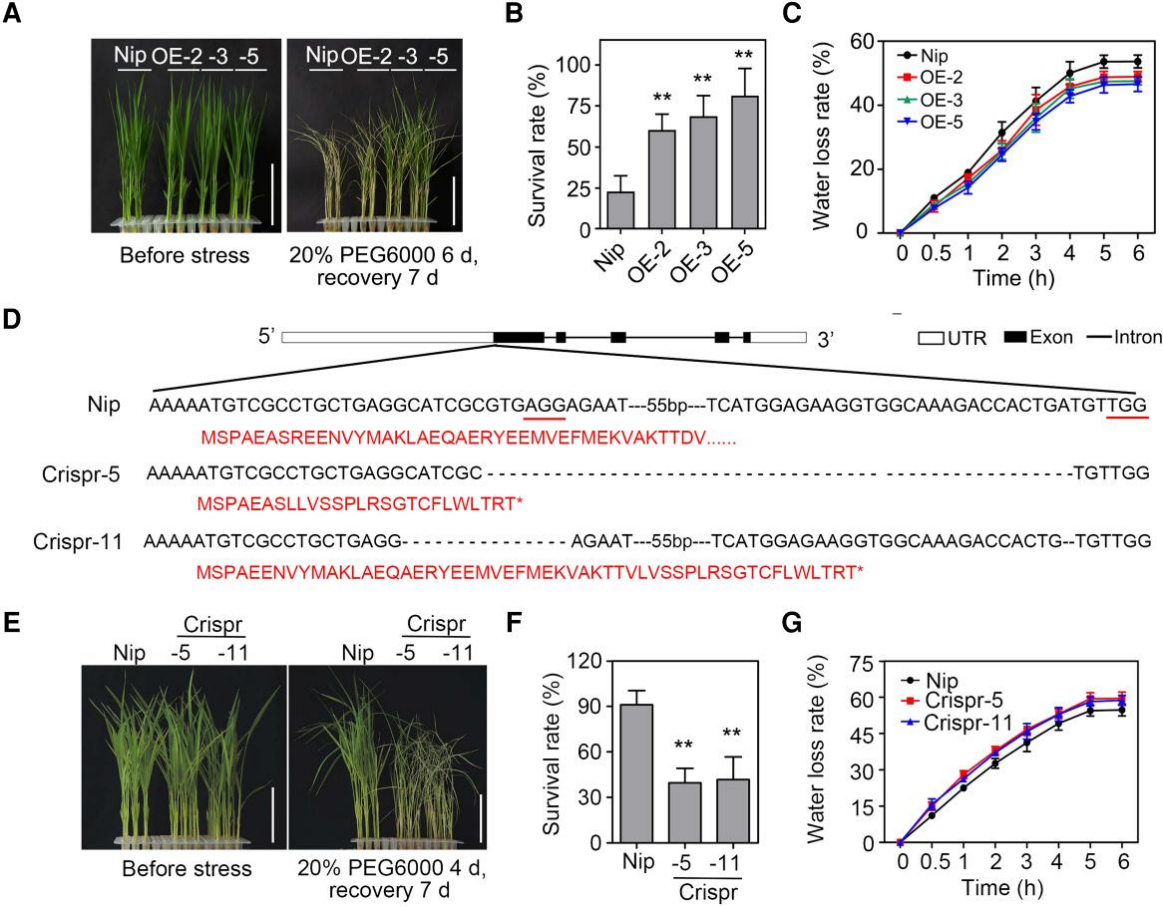
OsGF14f positively regulates osmotic stress tolerance in rice. **A)** Phenotypes of Nip and *OsGF14f*-OE seedlings upon osmotic stress treatment. Two-week-old seedlings were treated with 20% PEG6000 (dehydration mimic) for 6 d and allowed to recover growth for 7 d before being photographed. Scale bars = 5 cm. OE, overexpression transgenic line. **B)** Survival rates of the osmotic stress-treated seedlings as shown in **A)**. Data represent means ± Sd of 3 biological replicates with 16 seedlings used for each replicate. **P < 0.01 (unpaired 2-tailed Student's *t* test). **C)** Water loss rate of detached leaves from 2-wk-old Nip and *OsGF14f*-OE seedlings. Data represent means ± Sd of 3 individual plants per genotype. UTR, untranslated regions. **D)** CRISPR/Cas9-mediated target mutagenesis of *OsGF14f*. The protospacer adjacent motifs (PAMs) in the native sequences are underlined. Deletions are shown as dashed lines. Red letters represent amino acid sequences. Asterisks indicate the termination of protein translation. Crispr, knockout transgenic line by CRISPR/Cas9 gene editing system. **E)** Phenotypes of Nip and *OsGF14f*-Crispr seedlings following osmotic stress treatment. Two-week-old seedlings were treated with 20% PEG6000 for 4 d and allowed to recover growth for 7 d before being photographed. Scale bars = 5 cm. **F)** Survival rates of the osmotic stress–treated seedlings as shown in **E)**. Data represent means ± Sd of 3 biological replicates with at least 16 seedlings used for each replicate. **P < 0.01 (unpaired 2-tailed Student's *t* test). **G)** Water loss rate of detached leaves of 2-wk-old Nip and *OsGF14f*-Crispr seedlings. Data represent means ± Sd of 3 individual plants per genotype.

In this study, 2-wk-old plants were subjected to a 20% PEG6000 treatment for 6 d and then allowed to recover growth for 7 d under normal conditions. As presented in Fig. 2A, the 3 *OsGF14f*-OE lines showed much better growth than Nip after stress treatment, as manifested by more green leaves after a 7-d recovery. The survival rates of all 3 OE lines were over 60%, which was significantly higher than that of Nip (23%) (Fig. 2B). We then measured the water loss rate of detached leaves of all OE lines, as this is a widely used index for evaluating drought and osmotic stress tolerance in plants (Hu et al. 2006). The water loss rates of the *OsGF14f*-OE plants were much lower than that of the Nip control (Fig. 2C), demonstrating their enhanced tolerance to osmotic stress.

To further confirm the regulatory function of *OsGF14f* in rice osmotic stress response, we generated loss-of-function mutants using the CRISPR-Cas9 gene editing system (Ma et al. 2015). As shown in Fig. 2D, we obtained 2 homozygous transgenic lines, Crispr-5 and Crispr-11, in which *OsGF14f* was knocked out by the deletion of 94 and 13 nucleotides in the coding region, respectively. This led to a frameshift and the premature termination of the predicted protein. Under normal growth conditions, these 2 mutants displayed no obvious phenotypic differences from Nip (Supplemental Fig. S3). In contrast to the *OsGF14f*-OE plants, the *OsGF14f*-Crispr plants were much more sensitive to osmotic stress than Nip controls (Fig. 2E). After a 4-d treatment with 20% PEG6000 and a 7-d recovery period, 90% of the Nip plants survived, while only 39% Crispr-5 and 42% Crispr-11 plants survived (Fig. 2F). Consistent with the survival rate discrepancy, the leaf water loss rates of Crispr-5 and Crispr-11 plants were higher than that of Nip at different time points (Fig. 2G).

Given that leaf water loss is mainly controlled by stomata (Verslues et al. 2006), we checked stomatal apertures of *OsGF14f* transgenic and Nip plants. As shown in Supplemental Fig. S4, A and B, the percentages of completely closed, completely open, and partially open stomata were not obviously different among different genotypes under normal conditions. However, after 20% PEG6000 treatment, 44.2% and 40.4% of stomata were completely closed, and 10.3% and 13.3% of stomata were completely open in Crispr-5 and Crispr-11 lines. Nip leaves showed 62.6% completely closed and 5.8% completely open stomata. However, OE-3 and OE-5 leaves showed 68.3% and 69.5%completely closed and 4.4% and 2.8% completely open stomata. Then, we measured stomatal conductance of osmotically stressed plants. The stomatal conductance was lower in OE plants, while higher in Crispr plants, when compared to those in Nip plants (Supplemental Fig. S4C). Together, these results indicate that *OsGF14f* does not affect the normal growth of transgenic plants but does positively regulate the osmotic stress tolerance response in rice.

### OsGF14f affects the expression of osmotic stress–responsive genes

**Figure 3.**
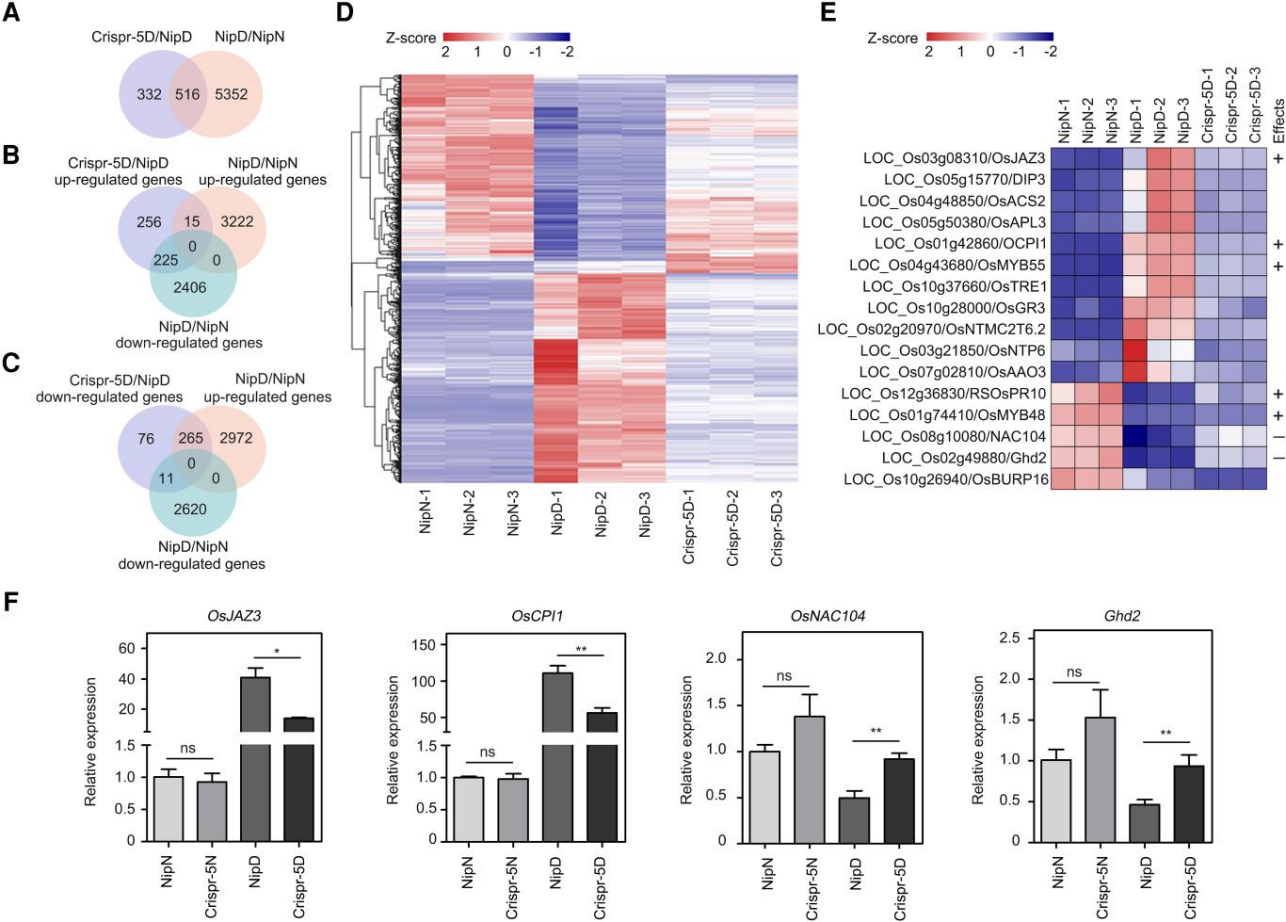
*OsGF14f* affects the expression of osmotic stress–responsive genes. Venn diagrams showing the genes regulated by OsGF14f and 20% PEG6000 treatment based on the RNA-Seq analysis. N, normal; D, 20% PEG6000. **B, C)** Venn diagrams showing the overlap of OsGF14f-regulated and osmotic stress–responsive genes. N, normal; D, 20% PEG6000. **D)** Heatmap showing the expression of (225 + 265) DEGs in Nip and *OsGF14f*-Crispr-5 plants. N, normal; D, 20% PEG6000. The heatmap was plotted using the OmicShare tools, a free online platform for data analysis (www.omicshare.com/tools) with Euclidean distance clustering algorithm. **E)** Heatmap showing the expression of 16 drought- and ABA-related DEGs in Nip and *OsGF14f*-Crispr-5 plants. “+” indicates genes that positively regulate drought tolerance; “−” indicates genes that negatively regulate drought tolerance. N, normal; D, 20% PEG6000.The heatmap was plotted using the OmicShare tools, a free online platform for data analysis (www.omicshare.com/tools) with Euclidean distance clustering algorithm. **F)** Validation of RNA-Seq data by RT-qPCR. Two-week-old Nip seedlings were exposed to a 20% PEG6000 treatment and harvested after 4 h for total RNA extraction and gene expression analysis. Each sample contained 5 uniform seedlings. Data represent means ± Sd of 3 independent experiments. *P < 0.05 and **P < 0.01 (unpaired 2-tailed Student's *t* test). ns, no significance; N, normal; D, 20% PEG6000; Nip, Nipponbare; Crispr, knockout transgenic line by CRISPR/Cas9 gene editing system.

To determine the molecular mechanisms of *OsGF14f* in rice osmotic stress tolerance, we performed RNA sequencing (RNA-Seq) assays to evaluate genome-wide gene expression differences between *OsGF14f*-Crispr seedlings and Nip plants under osmotic stress conditions. In Nip plants, after a 20% PEG6000 treatment, 5,868 differentially expressed genes (DEGs; defined as fold change [FC] > 2 and false discovery rate [FDR] < 0.05) (Fig. 3A, Supplemental Data Set 1) were identified, indicating the treatment is effective. Compared with Nip, 848 DEGs were found in Crispr-5 under osmotic stress conditions (Fig. 3A, Supplemental Data Set 2). Of these 848 DEGs, 496 and 352 genes were up- and downregulated, respectively (Supplemental Data Set 2). Among these DEGs, 516 (61%) were osmotic stress–responsive in Nip plants (Fig. 3A), suggesting that *OsGF14f* is required for the correct expression of numerous osmotic stress–responsive genes under osmotic stress conditions.

To further investigate how OsGF14f affects gene expression, we performed a clustering analysis using the 516 genes determined to be involved in osmotic stress response (Fig. 3, B to D). This analysis revealed that 225 of the upregulated genes (45%) in Crispr-5 plants showed downregulated expression patterns in Nip plants, while 265 downregulated genes (75%) in Crispr-5 plants showed upregulated expression patterns in Nip plants (Fig. 3, B to D). These results indicate that *OsGF14f* plays an important role in osmotic stress–responsive gene expression.

To further clarify the biological functions of the 516 osmotic stress–responsive DEGs, we retrieved the Molecular Breeding Knowledgebase (an integrated database that collected the known genes for rice, soybean [*Glycine max*], and wheat; https://www.mbkbase.org/) and found that 106 out of 516 DEGs have been functionally characterized (Supplemental Data Set 3). Interestingly, 84 genes were involved in various abiotic or biotic stress responses. Moreover, 16 genes are drought- and ABA-related genes, and 7 out of these 16 genes were functionally confirmed by transgenic assay (Fig. 3E). To check the reliability of RNA-Seq data, we selected 4 genes with known function (2 genes positively and 2 genes negatively regulate drought tolerance) for RT-qPCR verification. As shown in Fig. 3F, the expression trends of them were well consistent with the results obtained from the RNA-Seq analysis, indicating that the RNA-Seq data are reliable. Furthermore, the expression levels of the selective genes were comparable in Nip and *OsGF14f*-Crispr plants under normal conditions, indicating that *OsGF14f* did not affect their basal expression.

### OsGF14f positively regulates ABA response and interacts with OsbZIP23

**Figure 4.**
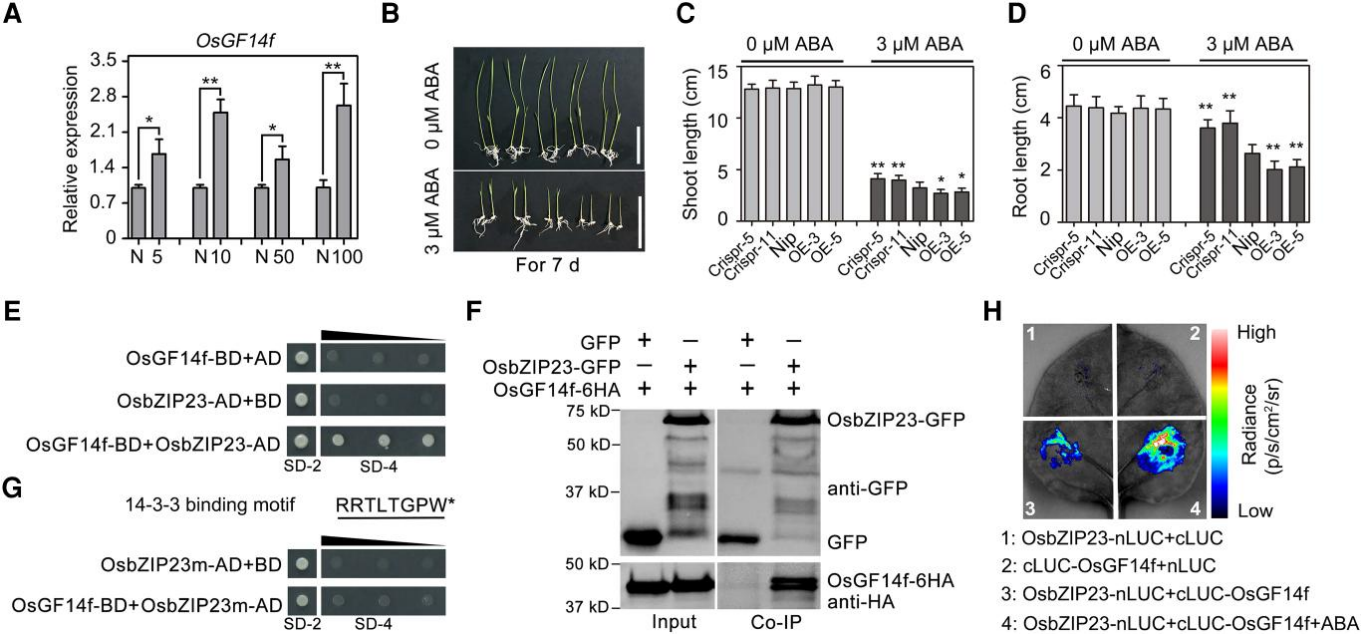
OsGF14f modulates rice ABA responses and interacts with OsbZIP23. **A)** RT-qPCR analysis showing *OsGF14f* expression following ABA treatment. Two-week-old Nip seedlings were treated with 5, 10, 50, and 100 *μ*m ABA or a mock substance for 12 h and then harvested for total RNA extraction for gene expression analysis. Each sample contained 5 uniform seedlings. Data represent means ± Sd of 3 independent experiments. *P < 0.05 and **P < 0.01 (unpaired 2-tailed Student's *t* test). N, normal. Numbers indicate the concentration of ABA. **B)** ABA sensitivity assay for Nip and *OsGF14f* transgenic plants. The seeds were germinated and grown on 1/2 MS medium plates containing either 0 or 3 *µ*m ABA for 7 d before being photographed. Scale bars = 5 cm. OE, overexpression transgenic line; Crispr, knockout transgenic line by CRISPR/Cas9 gene editing system. **C, D)** Statistical data of shoot length **C)** and root length **D)** as shown in **B)**. Data represent mean ± Sd of 13 individual plants per genotype. *P < 0.05 and **P < 0.01 (unpaired 2-tailed Student's *t* test). **E)** Y2H assay showing the interaction between OsGF14f and OsbZIP23. Protein interaction was determined by the growth of the yeast cells cotransformed with the indicated combinations of the plasmids on a synthetic dropout medium lacking Leu and Trp (SD-2) and a synthetic dropout medium lacking Leu, Trp, His, and adenine (SD-4). AD, pGADT7; BD, pGBKT7. Triangles represent the concentration gradients. **F)** Co-IP assay showing the interaction between OsGF14f and OsbZIP23 in vivo. GFP or OsbZIP23-GFP was transiently coexpressed in *N. benthamiana* leaves with OsGF14f-6HA, and Co-IP experiments using GFP-Trap were performed 3 d after *Agrobacterium* infiltration. Immunoblots were developed with anti-GFP antibodies to detect OsbZIP23 and with anti-HA to detect OsGF14f. **G)** Y2H assay depicting the interaction between OsGF14f and OsbZIP23m. The amino acid sequence of the 14-3-3 binding site located at the C-terminal end of OsbZIP23. Protein interaction was determined by the growth of the yeast cells cotransformed with various combinations of the plasmids on a synthetic dropout medium lacking Leu and Trp (SD-2) and a synthetic dropout medium lacking Leu, Trp, His, and adenine (SD-4). AD, pGADT7; BD, pGBKT7. Triangles represent the concentration gradients. **H)** LCI assay showing the interaction between OsGF14f and OsbZIP23 following ABA treatment in the leaves of *N. benthamiana*. Three days following infiltration, the indicated infiltrated regions were injected with 10 *µ*m ABA for 4 h before being photographed. Left: a representative leaf image. Right: the colored scale bar indicates luminescence intensity.

In plants, the hormone ABA plays a central role in the regulation of drought response (Zhu 2016). To test whether *OsGF14f* is involved in ABA signaling, we first determined its ability to respond to ABA treatment. The RT-qPCR results showed that the expression level of *OsGF14f* was significantly increased after treatment with different concentrations of ABA (Fig. 4A). Next, an ABA sensitivity assay was performed to further confirm the gene's involvement in ABA response. Without ABA treatment, *OsGF14f* transgenic plants and Nip displayed similar amounts of growth based on the shoot and root length (Fig. 4, B to D). Application of 3 *μ*m ABA inhibited the shoot and root growth of seedlings from all genotypes, but the extent of inhibition differed between the groups. When compared to Nip, the OE plants had shorter roots and shoots while the Crispr plants had longer ones (Fig. 4, B to D), indicating that OsGF14f positively regulates ABA-repressed seedling growth.

To get insights into the mechanism by which OsGF14f regulates osmotic stress tolerance in rice, we searched for proteins that interact with OsGF14f. With this aim, we screened a 2-wk-old rice seedling cDNA library using OsGF14f as the bait in the yeast 2-hybrid (Y2H) screen. In this assay, we identified 21 potential OsGF14f-interacting proteins (Supplemental Data Set 4). Among them, we were highly interested in OsbZIP23, which functions as a major transcription factor in the ABA signaling pathway (Xiang et al. 2008). So, we cloned the full-length *OsbZIP23* into prey vector and conducted Y2H experiment to verify the OsbZIP23–OsGF14f interaction (Fig. 4E). The interaction was further verified in *Nicotiana benthamiana* leaves by a coimmunoprecipitation (Co-IP) assay (Fig. 4F). In the Co-IP assay, OsbZIP23 was fused with a GFP tag (OsbZIP23-GFP), while OsGF14f was fused with a 6HA tag (OsGF14f-6HA). The OsGF14f-6HA was then coexpressed with either OsbZIP23-GFP or GFP alone in *N. benthamiana* leaves via *Agrobacterium* (*Agrobacterium tumefaciens*) infiltration. Following a Co-IP assay using GFP-Trap, it was found that the OsGF14f-6HA protein could be bound by OsbZIP23-GFP, but not by GFP, demonstrating the association of OsGF14f and OsbZIP23 in planta (Fig. 4F).

14-3-3 proteins generally interact with client proteins by using canonical 14-3-3 binding motifs to exert their regulatory function (Smith et al. 2011; Coblitz et al. 2006). By analyzing the protein sequence of OsbZIP23, we identified a canonical 14-3-3 binding motif (RRTLTGPW-COOH) at the C-terminal end (Fig. 4G). To test whether this 14-3-3 binding motif is responsible for the interaction between OsbZIP23 and OsGF14f, we generated an AD-OsbZIP23m vector lacking the 14-3-3 binding motif and performed a Y2H experiment. As shown in Fig. 4G, OsbZIP23m failed to interact with OsGF14f in yeast (*Saccharomyces cerevisiae*) cells, indicating that the 14-3-3 binding motif is necessary for OsbZIP23–OsGF14f interaction.

To further confirm the OsbZIP23–OsGF14f interaction and determine the effects of ABA on this interaction, we performed a firefly luciferase complementation imaging (LCI) assay. No obvious fluorescent signal was detected when either the OsbZIP23-nLUC/cLUC or nLUC/cLUC-OsGF14f constructs were coexpressed in *N. benthamiana* leaf cells. However, a strong fluorescent signal appeared after coexpressing OsbZIP23-nLUC/cLUC-OsGF14f constructs (Fig. 4H), demonstrating that an interaction between OsbZIP23 and OsGF14f had occurred. Interestingly, a visibly enhanced fluorescent signal was observed in the leaf regions that had been treated with 10 *μ*m ABA, indicating that ABA promotes the OsbZIP23–OsGF14f interaction (Fig. 4H). Taken together, these results demonstrate that OsGF14f interacts with OsbZIP23 and plays a critical role in ABA signaling.

### OsGF14f does not affect the protein stability of OsbZIP23

14-3-3 proteins have been reported to regulate the stability of their client proteins (Sirichandra et al. 2010; Chen et al. 2013; Liu et al. 2017). To explore this possible effect, we tested whether OsGF14f impacts the protein expression of OsbZIP23. First, we constructed *proUBQ10:OsbZIP23*-*GFP* overexpressing transgenic rice plants in Nip background (Supplemental Fig. S5A). We further crossed *OsbZIP23*-*GFP*-9 with *OsGF14f*-Crispr-5 and *OsGF14f*-OE-5 to generate *OsbZIP23*-*GFP*-9/*OsGF14f*-Crispr-5 and *OsbZIP23*-*GFP*-9/*OsGF14f*-OE-5 plants, respectively. We subjected the resulting 2-wk-old rice seedlings to 20% PEG6000 and 100 *μ*m ABA treatment for 24 h, before harvesting them for protein analysis. As shown in Supplemental Fig. S6, the levels of OsbZIP23-GFP protein were dramatically increased upon PEG6000 and ABA treatments; however, these protein levels were comparable in *OsbZIP23*-*GFP*-9, *OsbZIP23*-*GFP*-9/*OsGF14f*-Crispr-5, and *OsbZIP23*-*GFP*-9/*OsGF14f*-OE-5 plants under normal, PEG6000, and ABA treatment conditions. These data indicate that OsGF14f does not impact the protein stability of OsbZIP23.

### OsGF14f promotes the transcriptional regulation activity of OsbZIP23

**Figure 5.**
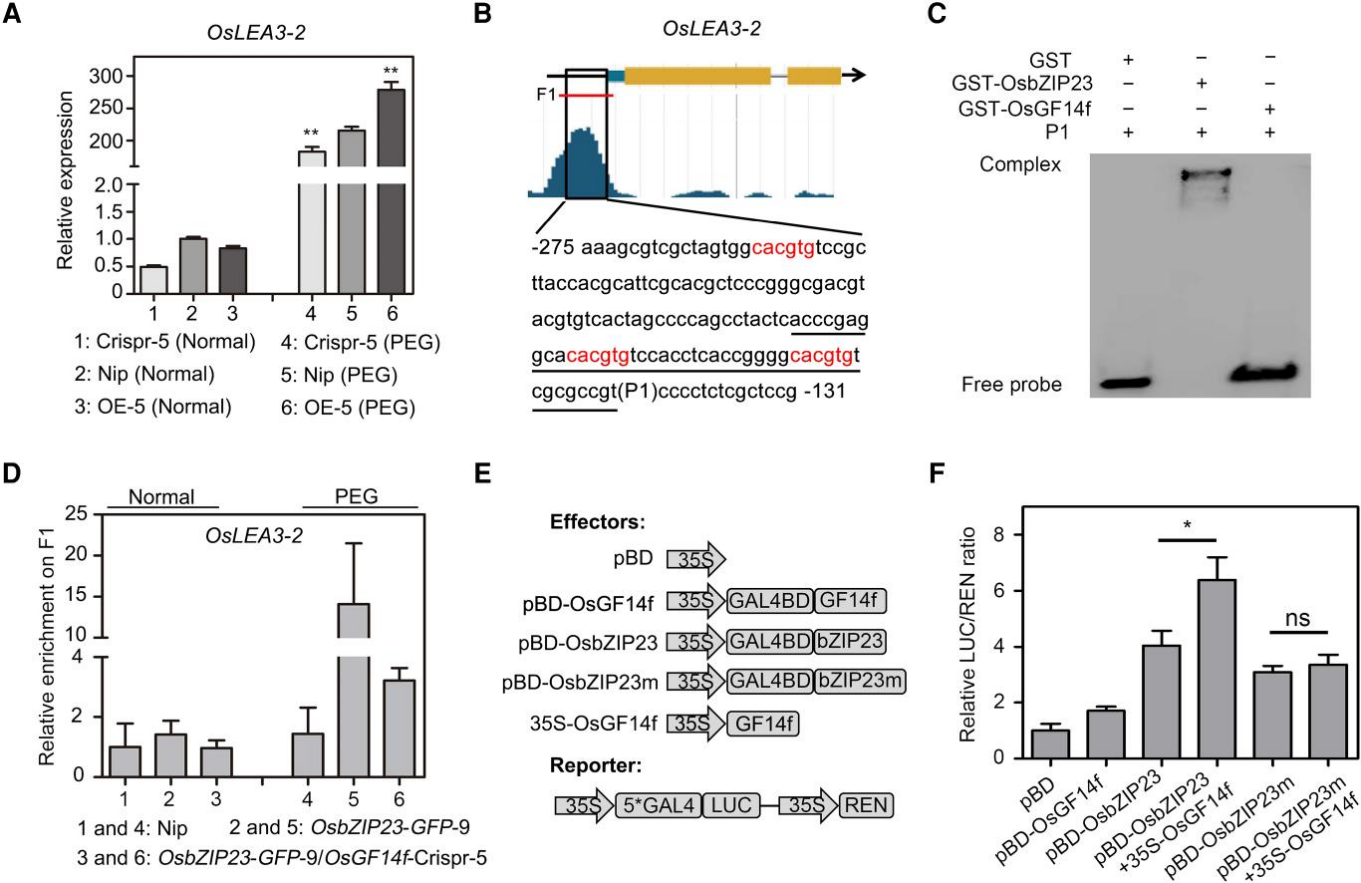
OsGF14f enhances the transcriptional regulation activity of OsbZIP23. **A)** RT-qPCR analysis showing *OsLEA3*-*2* expression in Nip and *OsGF14f* transgenic plants under normal and osmotic stress conditions. Two-week-old seedlings were treated with 20% PEG6000 or a mock substance for 4 h and then harvested for total RNA extraction and gene expression analysis. Each sample contained 5 uniform seedlings. Data represent means ± Sd of 6 independent experiments. **P < 0.01 (unpaired 2-tailed Student's *t* test). OE, overexpression transgenic line; Crispr, knockout transgenic line by CRISPR/Cas9 gene editing system. **B)** Schematic diagram showing the gene structure of *OsLEA3*-*2* and binding by OsbZIP23. The data were obtained from http://bioinfo.sibs.ac.cn/plant-regulomics/index.php. The G-box motifs are marked with red letters. The probe sequence (P1) for the EMSA is underlined. The F1 segment is used for ChIP-qPCR analysis shown in **D)**. **C)** EMSA showing the binding ability of OsbZIP23 toward the *OsLEA3*-*2* promoter (P1) in vitro. Glutathione S-transferase (GST) protein was used as a negative control. **D)** ChIP-qPCR analysis showing the in vivo binding of OsbZIP23 to the *OsLEA3*-*2* promoter (F1, shown in **B**) in Nip, *OsbZIP23*-*GFP*-9, and *OsbZIP23*-*GFP*-9*/OsGF14f*-Crispr-5 seedlings under normal and osmotic stress conditions. Two-week-old seedlings were treated with 20% PEG6000 or a mock substance for 4 h and then harvested for ChIP assay. Each sample contained 2 g seedlings. Data represent means ± Sd of 3 independent experiments. **E)** Diagram of constructs used for a transcription activity assay as shown in **F)**. pBD-OsbZIP23, pBD-OsGF14f, and 35S-OsGF14f were used as effectors. **F)** Transcription activity analysis showing the effect of OsGF14f on the transactivation activity of OsbZIP23. LUC/REN ratios represent the transcription regulation activities and were normalized to the activity of negative control pBD. Values are means ± Sd of 3 independent experiments. *P < 0.05 (unpaired 2-tailed Student's *t* test). ns, no significance.

It is well established that OsbZIP23 functions as a transcription factor that can directly bind to the G-box (CACGTG) to modulate downstream gene expression (Zong et al. 2016). Therefore, we first tested whether OsGF14f affects the DNA-binding activity of OsbZIP23. To do so, we selected the well-studied gene *OsLEA3*-*2* to use as an example for our investigation. Following a 20% PEG6000 treatment, when compared with Nip, *OsLEA3*-*2* showed a significantly higher expression in *OsGF14f*-OE-5, but lower in *OsGF14f*-Crispr-5 plants (Fig. 5A). A 46-bp DNA sequence, containing 2 G-box motifs in the OsbZIP23 binding region, was used as a probe for electrophoretic mobility shift assay (EMSA) (Fig. 5B). As expected, GST-OsbZIP23, but not GST or GST-OsGF14f proteins, could bind to the DNA probe, confirming the direct binding of ObZIP23 to *OsLEA3*-*2* (Fig. 5C). Accordingly, the results of chromatin immunoprecipitation (ChIP) quantitative PCR (ChIP-qPCR) assays showed that *OsbZIP23*-*GFP* could be highly enriched in the G-box–containing region (F1) of the *OsLEA3–2* promoter after 20% PEG6000 treatment (Fig. 5D). Interestingly, this enrichment was reduced in plants from an *OsGF14f*-Crispr-5 background, indicating that a functional OsGF14f is required for the effective binding of OsbZIP23 to its target gene *OsLEA3*-*2*. These results together suggest that although OsGF14f does not have DNA binding ability, it is required for the full binding of OsbZIP23 toward downstream target genes.

OsbZIP23 is known to have strong transcriptional activation activity (Xiang et al. 2008), so we used a dual-luciferase (LUC) reporter assay to examine if OsGF14f can alter this activity (Fig. 5, E and F). Consistent with previous studies (Xiang et al. 2008), our results indicated that both OsbZIP23 and OsbZIP23m (lacking the 14-3-3 binding motif) had obvious transcriptional activation activity (Fig. 5F). We also observed that OsGF14f showed weak transcriptional activation activity (Fig. 5F), similar to the 14-3-3 protein ATF1 in Arabidopsis (Wang et al. 1999). Interestingly, coexpressing *pro35S:OsGF14f* with pBD-OsbZIP23 resulted in a significantly higher LUC/REN ratio than that of pBD-OsbZIP23 alone (Fig. 5F), suggesting that the coexpression of OsGF14f could enhance the transcriptional activation activity of OsbZIP23. But the enhancement disappeared in pBD-OsbZIP23m pairs (Fig. 5F), indicating that the promotion of OsGF14f to the transcriptional regulation activity of OsbZIP23 depends on the OsGF14f–OsbZIP23 interaction.

### Mutation of *OsGF14f* largely attenuates the osmotic stress tolerance of *OsbZIP23*-*GFP* plants

**Figure 6.**
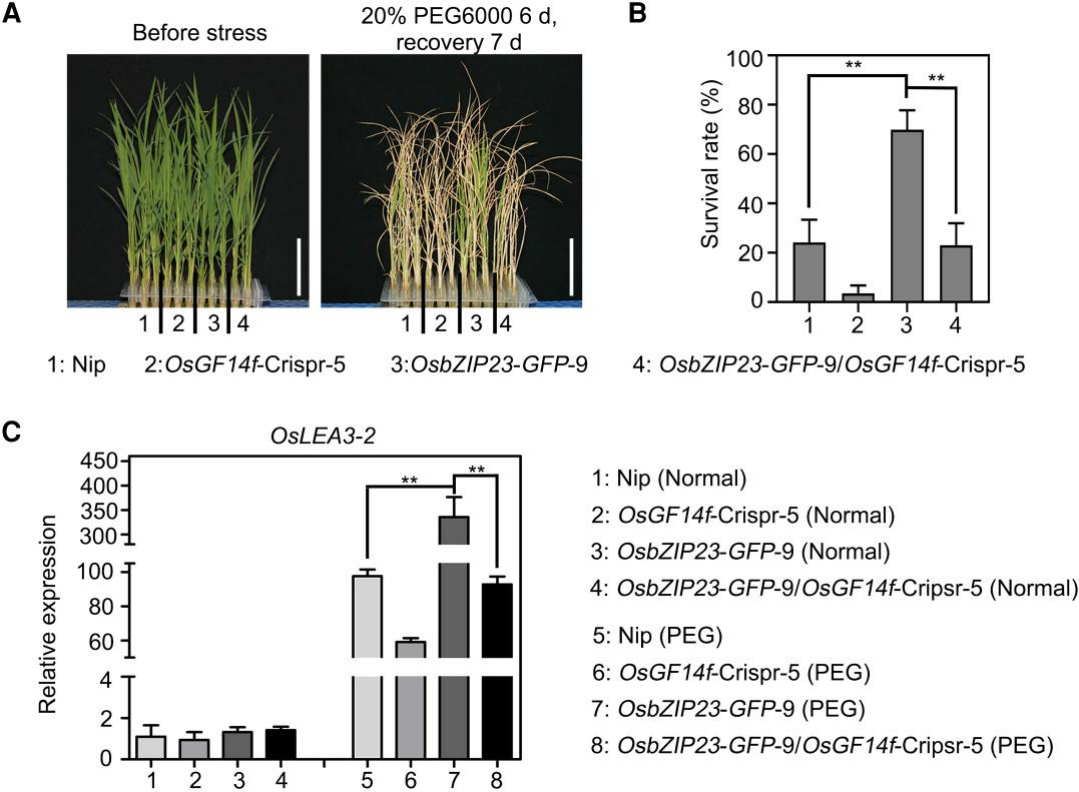
Mutation of *OsGF14f* attenuates the osmotic stress tolerance of *OsbZIP23*-*GFP* plants. **A)** Phenotypes of Nip, *OsGF14f*-Crispr-5, *OsbZIP23*-*GFP*-9, and *OsbZIP23*-*GFP*-9/*OsGF14f*-Crispr-5 plants following osmotic stress treatment. Two-week-old seedlings were treated with 20% PEG6000 for 6 d and allowed to recover growth for 7 d before being photographed. Crispr, knockout transgenic line by CRISPR/Cas9 gene editing system. Scale bars = 5 cm. **B)** Survival rates of the osmotic stress–treated seedlings as shown in **A)**. Data represent means ± Sd of 3 biological replicates with at least 16 seedlings used for each replicate. **P < 0.01 (unpaired 2-tailed Student's *t* test). **C)** RT-qPCR analysis of *OsLEA3*-*2* expression in Nip, *OsGF14f*-Crispr-5, *OsbZIP23*-*GFP*-9, and *OsbZIP23*-*GFP*-9/*OsGF14f*-Crispr-5 plants under normal and osmotic stress conditions. Two-week-old seedlings were treated with 20% PEG6000 or a mock substance for 4 h and then harvested for total RNA extraction and gene expression analysis. Each sample contained 5 uniform seedlings. Data represent means ± Sd of 3 independent experiments. **P < 0.01 (unpaired 2-tailed Student's *t* test).

Having ascertained that OsGF14f interacts with and affects the transcriptional regulatory activity of OsbZIP23 (Figs. 4 and 5), we sought to determine whether the full action of OsbZIP23 in osmotic stress response requires a functional OsGF14f. To test this, we compared the osmotic stress tolerance performances of *OsbZIP23*-*GFP*-9, *OsGF14f*-Cripsr-5, and *OsbZIP23*-*GFP*-9/*OsGF14f*-Crispr-5 plants. Consistent with results from previous studies (Xiang et al. 2008), *OsbZIP23*-*GFP*-9 plants exhibited an increased tolerance, while *OsGF14f*-Cripsr-5 plants showed a decreased tolerance to osmotic stress when compared to Nip (Fig. 6A). However, the *OsbZIP23*-*GFP*-9/*OsGF14f*-Crispr-5 plants displayed significantly weaker osmotic stress tolerance than *OsbZIP23*-*GFP*-9 plants (Fig. 6, A and B). After a 7-d recovery growth period, about 70% of the *OsbZIP23*-*GFP*-9 plants survived, whereas the *OsbZIP23*-*GFP*-9/*OsGF14f*-Crispr-5 plants were similar to the Nip control, with a survival rate of only 23% (Fig. 6B).

To determine the osmotic stress response at a molecular level, we used a RT-qPCR assay to analyze the *OsLEA3*-*2* expression in Nip, *OsbZIP23*-*GFP*-9, *OsGF14f*-Cripsr-5, and *OsbZIP23*-*GFP*-9/*OsGF14f*-Crispr-5 plants. Under normal conditions, the expression of *OsLEA3*-*2* had no significant differences among these genotypes examined (Fig. 6C). Upon a 20% PEG6000 treatment, the transcription of *OsLEA3*-*2* was increased in all lines, but when compared with Nip controls, it was higher in *OsbZIP23*-*GFP*-9 plants and lower in *OsGF14f*-Crispr-5 plants (Fig. 6C). Moreover, the expression level of *OsLEA3*-*2* was much lower in the *OsbZIP23*-*GFP*-9/*OsGF14f*-Crispr-5 plants than in the *OsbZIP23*-*GFP*-9 plants (Fig. 6C). Both the phenotypic and expression data demonstrate that OsGF14f is required for the full function of OsbZIP23 in the rice osmotic stress response.

### The increased osmotic stress tolerance of *OsGF14f*-OE plants also requires a functional OsbZIP23

**Figure 7.**
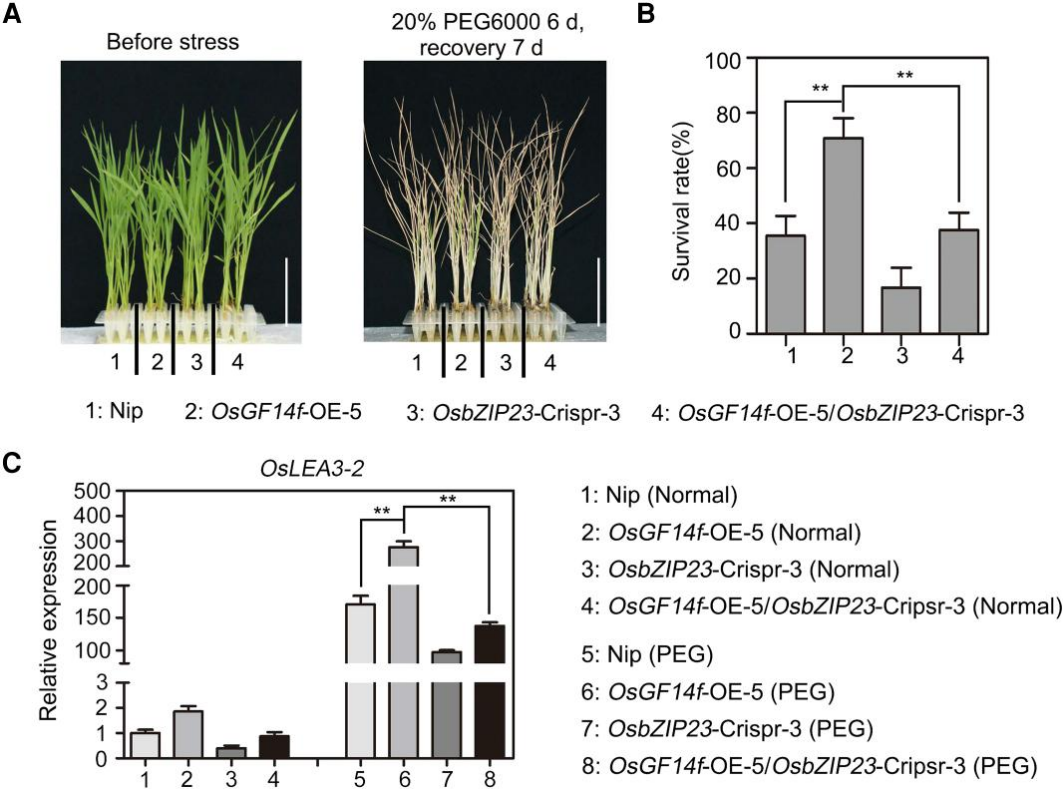
OsGF14f-mediated osmotic stress tolerance is partially dependent on OsbZIP23. **A)** Phenotypes of Nip, *OsGF14f*-OE-5, *OsbZIP23*-Crispr-3, and *OsGF14f*-OE-5/*OsbZIP23*-Crispr-3 plants following osmotic stress treatment. Two-week-old seedlings were treated with 20% PEG6000 for 6 d and allowed to recover growth for 7 d before being photographed. OE, overexpression transgenic line; Crispr, knockout transgenic line by CRISPR/Cas9 gene editing system. Scale bars = 5 cm. **B)** Survival rates of the osmotic stress–treated seedlings as shown in **A)**. Data represent means ± Sd of 3 biological replicates with 16 seedlings used for each replicate. **P < 0.01 (unpaired 2-tailed Student's *t* test). **C)** RT-qPCR analysis of *OsLEA3*-*2* expression in Nip, *OsGF14f*-OE-5, *OsbZIP23*-Crispr-3, and *OsGF14f*-OE-5/*OsbZIP23*-Crispr-3 plants under normal and osmotic stress conditions. Two-week-old seedlings were treated with 20% PEG6000 or a mock substance for 4 h and then harvested for total RNA extraction and gene expression analysis. Each sample contained 5 uniform seedlings. Data represent means ± Sd of 3 independent experiments. **P < 0.01 (unpaired 2-tailed Student's *t* test).

To investigate whether OsGF14f-regulated rice osmotic stress tolerance needs a functional OsbZIP23, we generated *OsbZIP23*-Crispr transgenic rice plants (Supplemental Fig. S5B). We then performed an osmotic stress tolerance assay using Nip, *OsGF14f*-OE-5, *OsbZIP23*-Crispr-3, and *OsGF14f*-OE-5/*OsbZIP23*-Crispr-3 transgenic plants. After a 6-d drought stress treatment and a 7-d recovery, the survival rates of Nip, *OsGF14f*-OE-5, *OsbZIP23*-Crispr-3, and *OsGF14f*-OE-5/*OsbZIP23*-Crispr-3 seedlings were about 35%, 71%, 17%, and 38%, respectively (Fig. 7, A and B). Next, we assessed the transcript levels of all tested plants by RT-qPCR assay. We found that the transcript level of *OsLEA3*-*2* was significantly higher in *OsGF14f*-OE-5 plants than that in Nip after the 20% PEG6000 treatment. Moreover, the expression level of *OsLEA3*-*2* was much lower in *OsGF14f*-OE-5/*OsbZIP23*-Crispr-3 transgenic plants than in that of *OsGF14f*-OE-5 plants (Fig. 7C). These results suggest that OsbZIP23 is partially related to OsGF14f-mediated osmotic stress tolerance in rice.

The rice genome encodes 89 OsbZIP proteins (Nijhawan et al. 2008), and a phylogenetic tree analysis has revealed that OsbZIP23 and 10 other OsbZIPs were grouped into the A clade (Nijhawan et al. 2008; Lu et al. 2009) (Supplemental Fig. S7A). Interestingly, most members in this clade contain a canonical 14-3-3 binding motif at the C-terminal end (Supplemental Fig. S7A). This prompted us to investigate whether these OsbZIPs also interact with OsGF14f. To test this, we chose basic leucine zipper 46 (OsbZIP46) and basic leucine zipper 72 (OsbZIP72) as examples to perform a Y2H assay and observed their interaction with OsGF14f in yeast cells (Supplemental Fig. S7B). Similar to OsbZIP23, OsbZIP46 and OsbZIP72 have also been functionally confirmed as positive regulators of drought tolerance response in rice (Lu et al. 2009; Tang et al. 2012); thus, they may be a functional redundancy of OsbZIP23 in OsGF14f-regulated osmotic stress response in rice.

### *OsGF14f* is a target gene of *OsbZIP23*

**Figure 8.**
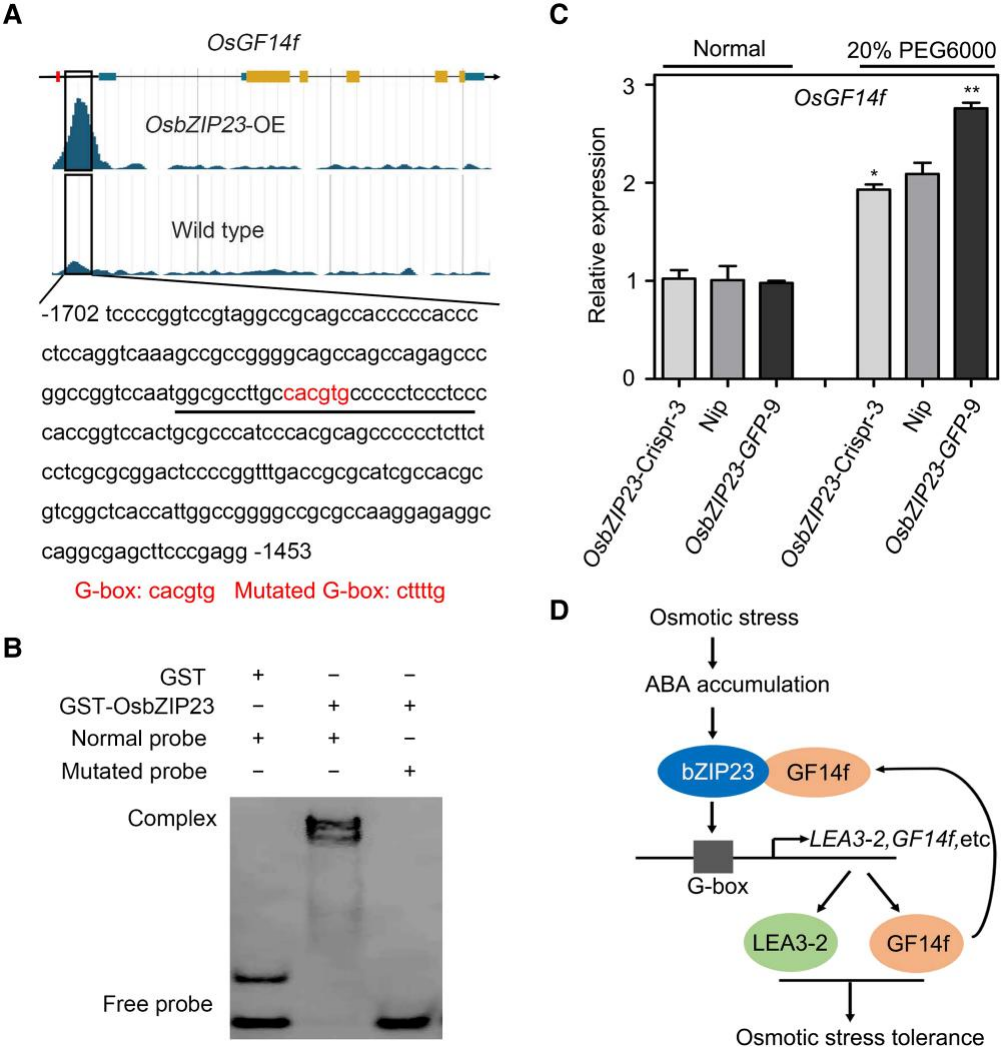
*OsGF14f* is a direct target gene of OsbZIP23. **A)** Schematic diagram showing the gene structure of *OsGF14f* and the binding by OsbZIP23. The data were retrieved and downloaded from http://bioinfo.sibs.ac.cn/plant-regulomics/index.php. The red box represents the location of the other G-box motif. The probe sequence for the EMSA is underlined. The G-box motif and its mutant version are shown at the bottom of the image. OE, overexpression transgenic line. **B)** EMSA showing the in vitro binding of OsbZIP23 toward the *OsGF14f* promoter. Glutathione S-transferase (GST) protein was used as a negative control. **C)** RT-qPCR analysis showing *OsGF14f* expression in Nip, *OsbZIP23*-Crispr-3, and *OsbZIP23*-*GFP*-9 plants under normal and osmotic stress conditions. Two-week-old seedlings were treated with 20% PEG6000 or a mock substance for 4 h and then harvested for total RNA extraction and gene expression analysis. Each sample contained 5 uniform seedlings. Data represent means ± Sd of 3 independent experiments. *P < 0.05 and **P < 0.01 (Student's *t* test). Crispr, knockout transgenic line by CRISPR/Cas9 gene editing system. **D)** A working model regarding the roles of OsbZIP23–OsGF14f module in rice osmotic stress response. Upon osmotic stress caused by drought, plan cells rapidly accumulate ABA, which enhanced the formation of the OsGF14f/OsbZIP23 complex, which binds to the G-box *cis*-elements in the promoter regions of stress-responsive genes. Drought-activated OsbZIP23 can also directly target *OsGF14f* to increase its expression, generating a positive feedback loop to promote the formation of the OsGF14f/OsbZIP23 complex.

To further explore the function relevance between OsGF14f and OsbZIP23, we analyzed the promoter sequence of *OsGF14f* and found 2 G-box motifs (CACGTG) (Fig. 8A). This prompted us to test whether *OsGF14f* is a direct target gene of OsbZIP23. First, we analyzed the previously published ChIP-Seq data of OsbZIP23 (Zong et al. 2016). As shown in Fig. 8A, a distinctive peak was detected approximately 1.5 kb upstream of the ATG start codon of *OsGF14f* in *OsbZIP23*-OE samples. We then selected a 27-bp sequence around this peak, containing the first G-box as a DNA probe, to conduct an EMSA. The results showed that OsbZIP23 could bind to the probe in a G-box–dependent manner (Fig. 8B).

We also found that OsbZIP23 positively regulated the osmotic stress–induced expression of *OsGF14f* (Fig. 8C). The transcript levels of *OsGF14f* were similar among Nip, *OsbZIP23*-*GFP*-9, and *OsbZIP23*-Crispr-3 plants under normal conditions. However, under a 20% PEG6000 treatment, the *OsGF14f* transcripts were much higher in the *OsbZIP23*-*GFP*-9 lines but lower in the *OsbZIP23*-Crispr-3 lines (Fig. 8C) compared to the Nip control, indicating that OsbZIP23 is an activator of *OsGF14f* under osmotic stress conditions. Collectively, these results demonstrate that *OsGF14f* is a target gene of OsbZIP23.

## Discussion

In this study, we showed that the 14-3-3 protein OsGF14f is a positive regulator of rice osmotic stress tolerance, and we explored the underlying molecular mechanisms. When plants encounter an osmotic stress, the endogenous ABA accumulates rapidly, leading to the increased transcription of *OsGF14f* and *OsbZIP23*. OsGF14f proteins interact with OsbZIP23 and enhance its transcriptional regulatory function to activate the expression of downstream stress-responsive genes (e.g. *LEA3*-*2*). Additionally, OsbZIP23 can directly target to and activate *OsGF14f*, enhancing its function and generating a positive feedback loop, thus conferring better tolerance to osmotic stress (Fig. 8D).

### OsGF14f interacts with OsbZIP23 and enhances its transcriptional activity

Based on prior studies, it has generally been believed that 14-3-3 proteins exert functional changes by affecting the action of their client proteins (de Boer et al. 2013; Zhao et al. 2021). Three canonical 14-3-3 binding motifs in client proteins have been defined so far: mode I RSXpS/pTXP, mode II RXF/YXpS/pTXP, and a C-terminal mode III motif p(S/T) X_1-2_-COOH (Coblitz et al. 2006; Smith et al. 2011). All are necessary for the full function of client proteins. For example, a mode III 14-3-3 binding motif is present at the C-terminus of ABRE BINDING FACTOR 3 (ABF3), which is required for interaction with 14-3-3 proteins and self-stability (Sirichandra et al. 2010; Chen et al. 2013). In our study, we found that OsGF14f interacts with OsbZIP23, and this interaction depends on the C-terminal mode III motif (Fig. 4, E to G). However, immunoblotting results showed that OsGF14f does not affect the stability of OsbZIP23 under normal, drought, and ABA treatment conditions (Supplemental Fig. S6).

Previously, 14-3-3 plant proteins were found to be part of a DNA binding complex that can associate with the G-box in vivo. However, it is known that 14-3-3 proteins do not have DNA binding ability in vitro (Lu et al. 1992; Schultz et al. 1998). How the 14-3-3 proteins take part in this transcription regulation complex remains unknown. In this study, we showed that OsGF14f plays important roles in the OsbZIP23-mediated transcription regulation machinery, in which OsGF14f is required for the full DNA binding activity of OsbZIP23 to the *OsLEA3*-*2* promoter (Fig. 5D). Furthermore, a dual-luciferase reporter assay showed that OsGF14f has a weak transcriptional activation activity but can promote that of OsbZIP23 (Fig. 5F). Consistent with this, the induced expression of *OsLEA3*-*2* was largely compromised in plants with an *OsGF14f*-Crispr background (Figs. 5A and 6C). Further genetic analysis showed that a disruption of OsGF14f can largely attenuate the osmotic stress–tolerant phenotype of the *OsbZIP23*-*GFP* plants (Fig. 6), confirming the critical role of OsGF14f in the OsbZIP23-mediated stress response. Collectively, these results provide support for our conclusion that OsGF14f interacts with OsbZIP23 and enhances its transcriptional activity, making it crucial for rice osmotic stress tolerance.

### OsGF14f-mediated osmotic stress tolerance is partially dependent on OsbZIP23

An osmotic stress tolerance test showed that the depletion of OsbZIP23 could decrease the tolerance of *OsGF14f*-OE plants, but the *OsGF14f*-OE-5/*OsbZIP23*-Crispr-3 plants had a much better performance than *OsbZIP23*-Crispr-3 plants (Fig. 7). This indicates that OsbZIP23 only partially contributes to OsGF14f-mediated osmotic stress tolerance in rice. Phylogenetic analysis showed that OsbZIP23, OsbZIP46, and OsbZIP72 are classified into the group A bZIPs (Supplemental Fig. S7A). The Y2H assay showed that OsbZIP46 and OsbZIP72 also interact with OsGF14f (Supplemental Fig. S7B), indicating the function of these OsbZIPs may be redundant in OsGF14f-mediated osmotic stress response.

Similar to OsbZIP23, OsbZIP72 could also bind to the ABRE and transactivate the downstream stress-responsive genes, and overexpression of *OsbZIP72* in plants resulted in enhanced drought tolerance (Lu et al. 2009). Overexpression of a constitutively active form of OsbZIP46 (OsbZIP46CA1, with a deletion of domain D) also led to increased drought tolerance (Tang et al. 2012). Transcriptomic analysis revealed that the overlapping of differentially regulated genes between OsbZIP46CA1 and OsbZIP23 was less than 25%. OsbZIP46CA1 can also regulate a different set of genes than OsbZIP23 (Tang et al. 2012). These findings suggest that OsGF14f may also regulate the transcription of OsbZIP46/72-dependent genes to confer stronger osmotic stress tolerance. This is most likely why a mutation of OsbZIP23 only partially, but not completely, disturbed the increased osmotic stress tolerance of *OsGF14f*-OE plants (Fig. 7, A and B).

### OsbZIP23 can bind to *OsGF14f* and induce feedback regulation

In this study, we have confirmed that OsGF14f can interact with OsbZIP23 to positively regulate drought-induced osmotic stress tolerance in rice. Interestingly, we also found that the *OsGF14f* promoter has 2 G-box motifs, which are the binding sites of OsbZIP23 (Fig. 8A), implying that OsbZIP23 can bind to *OsGF14f*. Both the ChIP-Seq data analysis (Fig. 8A) and the EMSA experiment (Fig. 8B) confirmed the binding compatibility of OsbZIP23 to *OsGF14f*. Furthermore, under osmotic stress conditions, the expression level of *OsGF14f* was significantly lower in the *OsbZIP23*-Crispr-3 lines but higher in *OsbZIP23*-*GFP*-9 lines than that in Nip plants (Fig. 8C). Thus, OsbZIP23 can bind to *OsGF14f* and positively regulate its expression. These results together suggest that the positive regulation of OsGF14f to *OsbZIP23* and the feedback regulation of OsbZIP23 to *OsGF14f* modulate the ABA-mediated osmotic stress response in rice (Fig. 8D). Similar results were reported in a previous study (Zong et al. 2016). OsbZIP23 is known to directly and positively regulate the expression level of *OsPP2C49* (encoding a PP2C protein negatively regulates ABA signaling) and *OsNCED4* (encoding a key enzyme involves in ABA biosynthesis) to generate a feedback regulation of ABA signaling in response to drought stress (Zong et al. 2016).

In summary, we have confirmed the positive regulatory roles of OsGF14f in rice osmotic stress tolerance. Our results suggest that the positive regulation of OsGF14f to OsbZIP23 and the feedback regulation of OsbZIP23 to OsGF14f modulate ABA-mediated osmotic stress response in rice. We have also observed that *OsGF14f* transgenic lines had no obvious change in the crucial agronomic traits including yield and plant height. Thus, our study provides insight into the regulatory mechanisms of rice in response to osmotic stress and offers a promising target for drought-tolerant rice breeding.

## Materials and methods

### Plant materials and growth conditions

The rice (*O. sativa*) *japonica* cultivar Nip was used in this study. Rice seeds were naturally air-dried and oven incubated at 49 °C for 3 d to break dormancy. Then, rice seeds were soaked in tap water for 2 d and allowed 2 d for germination at 30 °C. Germinated seeds were pregrown in a 1/4 Kimura B nutrient solution for 5 d and then in a 1/2 Kimura B nutrient solution for another 7-d period. The 2-wk-old seedlings were then treated in a 1/2 Kimura B nutrient solution containing 20% PEG6000 (*w*/*v*). All rice plants were grown in a 30 °C greenhouse with a 10-h light/14-h dark cycle (200 *µ*mol photons m^−2^ s^−1^). Arabidopsis (*A. thaliana*) ecotype Columbia-0 (Col-0) was grown in a 22 °C plant growth chamber with a 16-h light/8-h dark cycle (120 *µ*mol photons m^−2^ s^−1^). *Nicotiana benthamiana* plants were grown in a 25 °C plant growth chamber with a 16-h light/8-h dark cycle (120 *µ*mol photons m^−2^ s^−1^).

### Osmotic stress treatment and tissue sampling

For the osmotic stress tolerance assay, the seedlings were treated with 20% PEG6000 and allowed to recover for 7 d. Seedlings were regarded as survivors if healthy and young green leaves emerged after recovery. Three biological replicates (at least 16 seedlings for each biological replicate) were used. For the gene expression analysis, the seedlings were sampled after treatment over a series of time points. Each sample contained 5 uniform seedlings. All harvested samples were rapidly frozen in liquid nitrogen and stored at −80 °C for further RNA extraction.

### Plasmid construction and rice transformation

To generate the *OsGF14f* overexpression vector, we obtained the full-length coding region of the gene from Nip leaf cDNA by using PCR GF14f-OE-F/R primers and cloned the fragment into the PHQSN (modified from pCAMBIA1390) digested by Sal1 and Spe1 (Liu et al. 2016a). To create the *proUBQ10:OsbZIP23*-*GFP* and *proUBQ10:OsGF14f*-*GFP* vector, the full-length coding sequences of *OsbZIP23* and *OsGF14f* were PCR-amplified and cloned into the pCAMBIA1300-GFP vector by homologous recombination (Ma, Dong et al. 2022). To create the CRISPR constructs, PCR products containing 2 sgRNAs of *OsbZIP23* and *OsGF14f* were digested and inserted into a pYLCRISPR/Cas9 vector (Ma et al. 2015). The primers for creating the above constructs are listed in Supplemental Data Set 5. Transgenic rice plants were generated through *A. tumefaciens* (EH105)–mediated rice transformation by Wuhan Biorun Biosciences Co., Ltd (Wuhan, China). The details of mutants or transgenic lines are presented in Supplemental File 1.

### Water loss rate assay, stomatal conductance measurements, and scanning electron microscopy analysis of stomata

For the measurement of water loss, leaves were collected from Nip and *OsGF14f* transgenic plants grown in a 1/2 Kimura B nutrient solution for 2 wk. Immediately after detachment, each leaf was weighed on a piece of weighing paper and placed in an oven at 30 °C, with additional weighing at the designated time intervals. Three individual plants per genotype were performed, and the water loss rate was calculated by lost weight/the initial weight of the plants × 100%. For the stomatal conductance measurement, the second fully expanded leaves were applied to measure stomatal conductance with a portable gas analysis system (LI-6400XT, LI-COR, USA). For scanning electron microscopy (SEM) analysis, leaves of 2-wk-old Nip and *OsGF14f* transgenic plants with 20% PEG6000 treatment for 24 h or normal growth were detached and immediately fixed with 2.5% glutaraldehyde. Then the pictures of stomata were acquired by a SEM (S-3400N, Hitachi, Japan). The percentages of stomatal completely open, partially open, and completely close were calculated.

### ABA sensitivity assay

Nip and *OsGF14f* transgenic seeds were surface-sterilized and planted on 1/2 MS solid medium plates containing either 0 or 3 *μ*m ABA. Plates were grown in a 12-h light (28 °C)/12-h dark (26 °C) photoperiod. The shoot and root lengths were measured after 7 d of ABA treatment.

### RNA isolation and RT-qPCR analyses

Total RNA was extracted using the Hipure plant RNA Mini Kit (Magen, China). The RNA was then reverse transcribed into cDNA using the Primescript RT reagent Kit with gDNA Eraser (Perfect Real Time) (TaKaRa, China) according to the manufacturer's instructions. TB Green Premix Ex Taq II (Takara, China) was used for RT-qPCR analysis. The following PCR program on a CFX Connect real-time PCR detection system (Bio-Rad, USA) was used: 95 °C for 2 min, 45 cycles of 95 °C for 10 s, and 60 °C for 15 s, followed by a melting curve program. The rice *Ubiquitin* (*UBQ*) gene was used as an internal control. Relative expression levels of genes were calculated using the 2^−△△CT^ method (Livak and Schmittgen 2001). Primers used for RT-qPCR are listed in Supplemental Data Set 5.

### RNA-Seq assays

For RNA-Seq analysis, 2-wk-old Nip and *OsGF14f*-Crispr-5 seedlings were harvested at 4 h after a 20% PEG6000 treatment to extract total RNA with 3 biological replicates. For each replicate, 5 uniform seedlings for each genotype were used. The RNA samples were then sequenced on an Illumina NovaSeq 6000 platform and analyzed by Wuhan Metware Biotechnology Co., Ltd (Wuhan, China). DEGs were selected using DESeq2 (Love et al. 2014; Varet et al. 2016) with a relative change threshold of 2-fold (FDR < 0.05). Venny (https://bioinfogp.cnb.csic.es/tools/venny/) was used to perform the comparisons of DEGs. The heatmaps of gene expression profiles were generated on the OmicShare tools platform (http://www.omicshare.com/tools).

### Y2H screening

A Y2H screening was performed according to Matchmaker Yeast Two-Hybrid System (Clontech, USA). The cDNA library from the 2-wk-old rice seedlings of Nip was cloned as prey in plasmid vector pGADT7 using SMART cDNA Library Construction Kit (Clontech, USA, Code No. 634901). The coding region of *OsGF14f* was cloned into the pGBKT7 vector and used as the bait. Yeast (*S. cerevisiae*) transformants (strain Y187) were exhaustively selected on SD/-Ade/-His/-Leu/-Trp/X-a-Gal/AbA according to the manufacturer's instructions (Clontech, USA). The gene identities of all isolated positive plasmids were confirmed by DNA sequence analysis. The screening experiments were done by Guangzhou Ruizhen Biotechnology Co., Ltd (Guangzhou, China). The interaction between the OsGF14f and interactor candidates was reconfirmed with a targeted Y2H assay.

### Targeted Y2H assay

The coding sequence regions of *OsbZIP23*, *OsbZIP23m* (lacking the 14-3-3 binding motif), *OsbZIP46*, and *OsbZIP72* were each ligated into the pGADT7 vector by homologous recombination. The full length of *OsGF14f* was ligated into the pGBKT7 vector by homologous recombination. Yeast transformation was completed according to the manufacturer's instructions (Clontech, USA). The bait and prey vectors were cotransformed into the yeast strain AH109 and cocultured on an SD medium lacking Leu and Trp. After 3 to 4 d of incubation at 30 °C, the yeast cells were spotted in 10-, 100-, and 1000-fold dilutions on selection plates containing an SD medium lacking Leu, Trp, Ade, and His. These plates were further incubated at 30 °C until the yeast cells formed colonies. All primers used are listed in Supplemental Data Set 5.

### Co-IP assay

The full-length coding sequence of *OsbZIP23* was inserted into the pCAMBIA13000-GFP vector by homologous recombination (Ma, Dong et al. 2022), while the coding sequence of *OsGF14f* was inserted into the pGreen-35S-6HA vector by homologous recombination (Ma, Dong et al. 2022). All primers used are listed in Supplemental Data Set 5. The recombinant constructs were transiently expressed in 5-wk-old *N. benthamiana* leaves by *Agrobacterium* infiltration (strain GV3101). Proteins were extracted with a Co-IP buffer (50 mm Tris-HCl [pH 8.0], 150 mm NaCl, 1 mm EDTA, 0.5% Triton X-100, 1 mm DTT, 1 mm PMSF, and 1× protease inhibitor cocktail). The extracted proteins were incubated with GFP-Trap magnetic beads (Chromotek, Germany) for 3 h at 4 °C. The beads were then washed with the Co-IP buffer 5 times and collected by centrifugation. Then, the beads were resuspended in a protein extraction buffer and vortexed on the highest speed for 3 min to disassociate the proteins. Proteins were separated by SDS–PAGE and detected with anti-GFP (ab290, Abcam, UK, 1:3,000) and anti-HA (ab9110, Abcam, UK, 1:3,000) antibodies. The original images of blots are presented in Supplemental File 2.

### Firefly LCI assay

The coding sequences of *OsGF14f* and *OsbZIP23* were inserted into the pCAMBIA1300-cLUC vector and pCAMBIA1300-nLUC vector by homologous recombination, respectively (Ma, Dong et al. 2022). All primers used are listed in Supplemental Data Set 5. These plasmids were transformed into *A.* tumefaciens strain GV3101 and infiltrated into 5-wk-old *N. benthamiana* leaves. Three days after infiltration, the luminescence activity was captured with the Tanon-5200 chemiluminescent imaging system (Tanon, China). For ABA treatment, 10 *μ*m ABA treatment was applied to *Agrobacterium*-infiltrated *N. benthamiana* leaves 4 h before imaging.

### Transcriptional activation assay

The transcriptional activation activity of OsbZIP23 was measured using a dual-luciferase reporter assay in Arabidopsis (*A. thaliana*) protoplasts (Yang et al. 2018). The coding sequences of *OsGF14f*, *OsbZIP23*, and *OsbZIP23m* were amplified and inserted into pBD vector by homologous recombination (Yang et al. 2018). All primers used are listed in Supplemental Data Set 5. 5*×GAL4-LUC* was used as the reporter. The coding sequences of *OsGF14f* were amplified and inserted into the pGreen vector to generate pro35S:OsGF14f by homologous recombination. pBD-OsGF14f, pBD-OsbZIP23, pBD-OsbZIP23m, and pro35S:OsGF14f were used as effector plasmids. The indicated constructs were transfected into Arabidopsis protoplasts. After incubation in darkness for 16 h, the firefly and *Renilla* luciferase activities were measured using a Dual-Luciferase Reporter Assay System kit (Promega, China) in a 96-microplate luminometer instrument.

### EMSA

The EMSA experiments were performed as previously described (Li et al. 2022). The full-length coding sequence of *OsbZIP23* was cloned into pGEX-4T-3 by homologous recombination and transformed into *Escherichia coli* strain Arctic-Express (DE3) to express GST-ObZIP23 protein. The full-length coding sequence of *OsGF14f* was cloned into pGEX-4T-3 by homologous recombination and transformed into *E. coli* strain BL21 (DE3) to expression GST-OsGF14f protein. The recombinant proteins were affinity purified using mag-beads GST fusion protein purification (Sangon Biotech, China). Oligonucleotide probes (OsLEA3-2, 5′-acccgaggcacacgtgtccacctcaccggggcacgtgtcgcgccgt-3′; OsGF14f, 5′-tggcgccttgccacgtgccccctccct-3′) were synthesized and labeled with biotin at the 5′ end by Sangon Biotech. EMSA was performed using the EMSA Kit (Viagene, China) according to the manufacturer’s instructions. Labeled probes were mixed with purified recombinant protein in a binding buffer at room temperature for 20 min. The reaction mixtures were subjected to a 6% (*w*/*v*) native polyacrylamide gel and then transferred onto a nylon membrane and detected using the EMSA kit.

### ChIP-qPCR assays

ChIP assays were performed as previously described (Gendrel et al. 2005; Yang et al. 2020). About 2 g 2-wk-old rice transgenic seedlings with either normal or 20% PEG6000 treatment were cross-linked with 1% formaldehyde. Cross-linking was stopped with 0.125 m glycine. Chromatin was isolated and fragmented by sonication. The chromatin complexes were incubated with anti-GFP magnetic beads (Smart-Lifesciences, China) for 3 h. After washing, the cross-linking of eluted samples was reversed by incubation at 65 °C overnight. The coprecipitated DNA was recovered and analyzed using a qPCR. The chromatin isolated before precipitation was used as an input control. All primers used for ChIP-qPCR are listed in Supplemental Data Set 5.

### Extraction and fractionation of proteins and immunoblotting

To extract total protein, 0.2 g seedlings were ground in liquid nitrogen and homogenized with 0.5 mL total protein extraction buffer (20 mm Tris-HCl [pH 7.5], 150 mm NaCl, 1 mm EDTA, 1% Triton X-100, and protease inhibitor cocktail tablets (Roche, Switzerland). After the samples were centrifuged at 14,000 × *g* for 15 min at 4 °C, the supernatant was collected as the total protein. To isolate nuclei and cytoplasmic fractions, 0.4 g seedlings were ground in liquid nitrogen and homogenized with 1 mL lysis buffer (20 mm Tris-HCl at pH 7.0, 250 mm sucrose, 25% [*v*/*v*] glycerol, 20 mm KCl, 2 mm EDTA, 2.5 mm MgCl_2_, 1% [*v*/*v*] Triton X-100, and 1× protease inhibitor cocktail). The homogenate was then filtered through a layer of wet Miracloth (Millipore, USA) and centrifuged at 1,400 × *g* for 10 min at 4 °C to pellet the nuclei. The cytoplasmic fraction was transferred to a new tube and centrifuged at 14,000 × *g* for 10 min at 4 °C and the supernatant was collected as the cytoplasmic protein. The nuclear pellet was washed 5 times in 1 mL washing buffer (20 mm Tris-HCl [pH 7.0], 25% (*v*/*v*) glycerol, 2.5 mm MgCl_2_, and 1× protease inhibitor cocktail) by gently pipetting and centrifuged at 1,400 × *g* for 10 min at 4 °C, and the supernatant was discarded. Subsequently, the nuclear pellet was resuspended in 100 *μ*L total protein extraction buffer. Fifteen microliters of the total proteins or cytoplasmic and nuclear fractions was subjected to immunoblot analysis using anti-GFP (ab290, Abcam, UK, 1:3,000), anti-H3 (ab1791, Abcam, UK, 1:5,000), and anti-FBPase (AS04043, Agrisera, Sweden, 1:5,000) antibodies, respectively.

### Phylogenetic tree construction

The full-length amino acid sequences of group A OsbZIP proteins were downloaded from RGAP database and used to do the alignment in ClustalW. The neighbor-joining tree was constructed using MEGA7.0 with 1,000 bootstrap replicates. The alignment file is provided in Supplemental File 3. The Newick format of the phylogenetic tree is provided in Supplemental File 4.

### Statistical analysis

GraphPad Prism 5.0 and Microsoft Office Excel were used for statistical analysis. The significance of difference was examined by Student's *t* test (*P < 0.05; **P < 0.01). Detailed statistical analysis data are shown in Supplemental Data Set 6.

### Accession numbers

Sequence data from this article can be found in Rice Genome Annotation Project Database (http://rice.uga.edu/): OsGF14a (LOC_Os08g37490), OsGF14b (LOC_Os04g38870), OsGF14c (LOC_Os08g33370), OsGF14d (LOC_Os11g34450), OsGF14e (LOC_Os02g36974), OsGF14f (LOC_Os03g50290), OsGF14g (LOC_Os01g11110), OsGF14h (LOC_Os11g39540), OsLEA3-2(LOC_Os03g20680), OsbZIP09 (LOC_Os01g59760), OsbZIP10 (LOC_Os01g64000), OsbZIP12 (LOC_Os01g64730), OsbZIP23 (LOC_Os02g52780), OsbZIP29 (LOC_Os03g20650), OsbZIP40 (LOC_Os05g36160), OsbZIP42 (LOC_Os05g41070), OsbZIP46 (LOC_Os06g10880), OsbZIP62 (LOC_Os07g48660), OsbZIP66 (LOC_Os08g36790), and OsbZIP72 (LOC_Os09g28310).

The transcriptome data have been deposited in the NCBI GEO repository (http://www.ncbi.nlm.nih.gov/geo) with the accession no. PRJNA913293.

## Supplementary Material

koad211_Supplementary_Data

## Supplemental data

The following materials are available in the online version of this article.

**Supplemental Figure S1.** Expression analysis of rice 14-3-3 family genes.

**Supplemental Figure S2.** The response of *OsGF14f* transcripts to osmotic stress treatment in roots.

**Supplemental Figure S3.** Phenotypic observation of *OsGF14f* transgenic plants and Nip under normal growth conditions.

**Supplemental Figure S4.** The regulation of *OsGF14f* on stomatal aperture and conductance under normal and osmotic stress conditions.

**Supplemental Figure S5.** Molecular characterization of *OsbZIP23* transgenic plants.

**Supplemental Figure S6.** OsGF14f does not affect the protein stability of OsbZIP23.

**Supplemental Figure S7.** OsGF14f interacts with OsbZIP46 and OsbZIP72.

**Supplemental Data Set 1.** List of the DEGs in Nip plants upon osmotic stress treatment.

**Supplemental Data Set 2.** List of the DEGs in *OsGF14f*-Crispr-5 plants when compared to Nip upon osmotic stress treatment.

**Supplemental Data Set 3.** List of the 106 functionally characterized DEGs.

**Supplemental Data Set 4.** List of potential OsGF14f interaction partners identified by Y2H screening.

**Supplemental Data Set 5.** Primers used in this study.

**Supplemental Data Set 6.** Summary of statistical analyses.

**Supplemental File 1.** Genetics module.

**Supplemental File 2.** The original images of blots for Fig. 4F.

**Supplemental File 3.** Multiple sequence alignment for Supplemental Fig. S7A.

**Supplemental File 4.** Newick format of the phylogenetic tree for Supplemental Fig. S7A.

## Dive Curated Terms

The following phenotypic, genotypic, and functional terms are of significance to the work described in this paper:

ABF3 Gramene: AT4G34000
ABF3 Araport: AT4G34000

ABI5 Gramene: At2g36270
ABI5 Araport: At2g36270

UBQ10 Gramene: At4g05320
UBQ10 Araport: At4g05320

GEO Gramene: https://www.ncbi.nlm.nih.gov/geo/
GEO Araport: https://www.ncbi.nlm.nih.gov/geo/

OsbZIP23 Gramene: LOC_Os02g52780
OsbZIP23 Araport: LOC_Os02g52780

OsLEA3-2 Gramene: LOC_Os03g20680
OsLEA3-2 Araport: LOC_Os03g20680

## References

[koad211-B1] Bai MY , Zhang LY, Gampala SS, Zhu SW, Song WJ, Chong K, Wang ZY. Functions of OsBZR1 and 14-3-3 proteins in brassinosteroid signaling in rice. Proc Natl Acad Sci U S A. 2007:104(34):13839–13844. 10.1073/pnas.0706386104 17699623 PMC1959469

[koad211-B2] Chen Y-T , Liu HX, Stone S, Judy C. ABA and the ubiquitin E3 ligase KEEP ON GOING affect proteolysis of the *Arabidopsis thaliana* transcription factors ABF1 and ABF3. Plant J. 2013:75(6):965–976. 10.1111/tpj.12259 23742014 PMC3823012

[koad211-B3] Coblitz B , Wu M, Shikano S, Li M. C-terminal binding: an expanded repertoire and function of 14-3-3 proteins. FEBS Lett. 2006:580(6):1531–1535. 10.1016/j.febslet.2006.02.014 16494877

[koad211-B4] de Boer AH , van Kleeff PJM, Gao J. Plant 14-3-3 proteins as spiders in a web of phosphorylation. Protoplasma 2013:250(2):425–440. 10.1007/s00709-012-0437-z 22926776

[koad211-B5] Denison FC , Paul A-L, Zupanska AK, Ferl RJ. 14-3-3 proteins in plant physiology. Semin Cell Dev Biol. 2011:22(7):720–727. 10.1016/j.semcdb.2011.08.006 21907297

[koad211-B6] Fàbregas N , Lozano-Elena F, Blasco-Escámez D, Tohge T, Martínez-Andújar C, Albacete A, Osorio S, Bustamante M, Riechmann JL, Nomura T, et al Overexpression of the vascular brassinosteroid receptor BRL3 confers drought resistance without penalizing plant growth. Nat Commun. 2018:9(1):4680. 10.1038/s41467-018-06861-3 30409967 PMC6224425

[koad211-B7] Gendrel AV , Lippman Z, Martienssen R, Colot V. Profiling histone modification patterns in plants using genomic tiling microarrays. Nat Methods. 2005:2(3):213–218. 10.1038/nmeth0305-213 16163802

[koad211-B8] Ho SL , Huang LF, Lu CA, He SL, Wang CC, Yu SP, Chen J, Yu SM. Sugar starvation- and GA-inducible calcium-dependent protein kinase 1 feedback regulates GA biosynthesis and activates a 14-3-3 protein to confer drought tolerance in rice seedlings. Plant Mol Biol. 2013:81(4-5):347–361. 10.1007/s11103-012-0006-z 23329372

[koad211-B9] Hu HH , Dai MQ, Yao JL, Xiao BZ, Li XH, Zhang QF, Xiong LZ. Overexpressing a NAM, ATAF, AND CUC (NAC) transcription factor enhances drought resistance and salt tolerance in rice. Proc Natl Acad Sci U S A. 2006:103(35):12987–12992. 10.1073/pnas.0604882103 16924117 PMC1559740

[koad211-B10] Lee SC , Luan S. ABA signal transduction at the crossroad of biotic and abiotic stress responses. Plant Cell Environ. 2012:35(1):53–60. 10.1111/j.1365-3040.2011.02426.x 21923759

[koad211-B11] Li X , Liao J, Bai H, Bei J, Li K, Luo M, Shen WJ, Yang C, Gao CJ. Arabidopsis flowering integrator SOC1 transcriptionally regulates autophagy in response to long-term carbon starvation. J Exp Bot. 2022:73(19):6589–6599. 10.1093/jxb/erac298 35852462

[koad211-B12] Liu Z , Jia Y, Ding Y, Shi Y, Li Z, Guo Y, Gong ZZ, Yang SH. Plasma membrane CRPK1-mediated phosphorylation of 14-3-3 proteins induce their nuclear import to fine-tune CBF signaling during cold response. Mol Cell. 2017:66(1):117–128.e5. 10.1016/j.molcel.2017.02.016 28344081

[koad211-B13] Liu JP , Sun XJ, Liao WC, Zhang JH, Liang JS, Xu WF. Involvement of OsGF14b adaptation in the drought resistance of rice plants. Rice. 2019:12(1):82. 10.1186/s12284-019-0346-2 31728660 PMC6856252

[koad211-B14] Liu Q , Yang JY, Zhang SH, Zhao JL, Feng AQ, Yang TF, Wang XF, Mao XX, Dong JF, Zhu XY, et al *OsGF14b* positively regulates panicle blast resistance, but negatively regulates leaf blast resistance in rice. Mol Plant Microbe Interact. 2016a:29(1):46–56. 10.1094/MPMI-03-15-0047-R 26467468

[koad211-B15] Liu Q , Yang JY, Zhang SH, Zhao JL, Feng AQ, Yang TF, Wang XF, Mao XX, Dong JF, Zhu XY, et al *OsGF14e* positively regulates panicle blast resistance in rice. Biochem Biophys Res Commun. 2016b:471(1):247–252. 10.1016/j.bbrc.2016.02.005 26851365

[koad211-B16] Liu Q , Zhang SH, Liu B. 14-3-3 proteins: macro-regulators with great potential for improving abiotic stress tolerance in plants. Biochem Biophys Res Commun. 2016c:477(1):9–13. 10.1016/j.bbrc.2016.05.120 27233603

[koad211-B17] Livak K , Schmittgen T. Analysis of relative gene expression data using real-time quantitative PCR and the 2^−△△CT^ method. Methods 2001:25(4):402–408. 10.1006/meth.2001.1262 11846609

[koad211-B18] Love MI , Huber W, Anders S. Moderated estimation of fold change and dispersion for RNA-seq data with DESeq2. Genome Biol. 2014:15(12):550. 10.1186/s13059-014-0550-8 25516281 PMC4302049

[koad211-B19] Lu G , DeLisle AJ, de Vetten NC, Ferl RJ. Brain proteins in plants: an *Arabidopsis* homolog to neurotransmitter pathway activators in part of a DNA binding complex. Proc Natl Acad Sci U S A. 1992:89(23):11490–11494. 10.1073/pnas.89.23.11490 1454838 PMC50577

[koad211-B20] Lu GJ , Gao CX, Zheng XN, Han B. Identification of OsbZIP72 as a positive regulator of aba response and drought tolerance in rice. Planta 2009:229(3):605–615. 10.1007/s00425-008-0857-3 19048288

[koad211-B21] Ma YM , Dong JF, Yang W, Chen L, Wu W, Li WH, Zhou L, Wang J, Chen JS, Yang TF, et al OsFLZ2 interacts with OsMADS51 to fine-tune rice flowering time. Development 2022:149(24):dev200862. 10.1242/dev.200862 36515165

[koad211-B22] Ma YM , Yang JY, Dong JF, Zhang SH, Yang W, Zhao JL, Yang TF, Chen L, Zhou L, Wang J, et al Overexpression of OsGF14f enhances quantitative leaf blast and bacterial blight resistance in rice. Int J Mol Sci. 2022:23(13):7440. 10.3390/ijms23137440 35806444 PMC9266906

[koad211-B23] Ma XL , Zhang QY, Zhu QL, Liu W, Chen Y, Qiu R, Wang B, Yang ZF, Li HY, Lin YR, et al A robust CRISPR/Cas9 system for convenient, highefficiency multiplex genome editing in monocot and dicot plants. Mol Plant. 2015:8(8):1274–1284. 10.1016/j.molp.2015.04.007 25917172

[koad211-B24] Nakashima K , Tran L-SP, Nguyen DV, Fujita M, Maruyama K, Todaka D, Ito Y, Hayashi N, Shinozaki K, Yamaguchi-Shinozaki K. Functional analysis of a NAC-type transcription factor OsNAC6 involved in abiotic and biotic stress-responsive gene expression in rice. Plant J. 2007:51(4):617–630. 10.1111/j.1365-313X.2007.03168.x 17587305

[koad211-B25] Nakashima K , Yamaguchi-Shinozaki K. ABA signaling in stress-response and seed development. Plant Cell Rep. 2013:32(7):959–970. 10.1007/s00299-013-1418-1 23535869

[koad211-B26] Nijhawan A , Jain M, Tyagi AK, Khurana JP. Genomic survey and gene expression analysis of the basic leucine zipper transcription factor family in rice. Plant Physiol. 2008:146(2):333–350. 10.1104/pp.107.112821 18065552 PMC2245831

[koad211-B27] Ray DK , Mueller ND, West PC, Foley JA, Hart JP. Yield trends are insufficient to double global crop production by 2050. PLoS One 2013:8(6):e66428. 10.1371/journal.pone.0066428 PMC3686737 23840465

[koad211-B28] Ray DK , Ramankutty N, Mueller ND, West PC, Foley JA. Recent patterns of crop yield growth and stagnation. Nat Commun. 2012:3(1):1293. 10.1038/ncomms2296 23250423

[koad211-B29] Schultz TF , Medina J, Hill A, Quatrano RS. 14-3-3 proteins are part of an abscisic acid–VIVIPAROUS1 (VP1) response complex in the Em promoter and interact with VP1 and EmBP1. Plant Cell 1998:10(5):837–847. 10.1105/tpc.10.5.837 9596641 PMC144375

[koad211-B30] Shao HB , Chu LY, Jaleel CA, Zhao CX. Water-deficit stress-induced anatomical changes in higher plants. C R Biol. 2008:331(3):215–225. 10.1016/j.crvi.2008.01.002 18280987

[koad211-B31] Sirichandra C , Davanture M, Turk BE, Zivy M, Valot B, Leung J, Merlot S. The Arabidopsis ABA-activated kinase OST1 phosphorylates the bZIP transcription factor ABF3 and creates a 14-3-3 binding site involved in its turnover. PLoS One 2010:5(11):e13935. 10.1371/journal.pone.0013935 PMC2978106 21085673

[koad211-B32] Smith AJ , Daut J, Schwappach B. Membrane proteins as 14-3-3 clients in functional regulation and intracellular transport. Physiology (Bethesda) 2011:26(3):181–191. 10.1152/physiol.00042.2010 21670164

[koad211-B33] Srivastava AK , Zhang C, Caine RS, Gray J, Sadanandom A. Rice SUMO protease Overly Tolerant to Salt 1 targets the transcription factor, OsbZIP23 to promote drought tolerance in rice. Plant J. 2017:92(6):1031–1043. 10.1111/tpj.13739 29024118

[koad211-B34] Tang N , Zhang H, Li XH, Xiao JH, Xiong LZ. Constitutive activation of transcription factor OsbZIP46 improves drought tolerance in rice. Plant Physiol. 2012:158(4):1755–1768. 10.1104/pp.111.190389 22301130 PMC3320183

[koad211-B35] Tardieu F . Any trait or trait-related allele can confer drought tolerance: just design the right drought scenario. J Exp Bot. 2012:63(1):25–31. 10.1093/jxb/err269 21963615

[koad211-B36] Varet H , Brillet-Guéguen L, Coppée J-Y, Dillies M-A. SARTools: a DESeq2- and EdgeR-based r pipeline for comprehensive differential analysis of RNA-seq data. PLoS One 2016:11(6):e0157022. 10.1371/journal.pone.0157022 PMC4900645 27280887

[koad211-B37] Verslues PE , Agarwal M, Katiyar-Agarwal S, Zhu J, Zhu JK. Methods and concepts in quantifying resistance to drought, salt and freezing, abiotic stresses that affect plant water status. Plant J. 2006:45(4):523–539. 10.1111/j.1365-313X.2005.02593.x 16441347

[koad211-B38] Wang J , Goodman HM, Zhang H. An *Arabidopsis* 14-3-3 protein can act as a transcriptional activator in yeast. FEBS Lett. 1999:443(3):282–284. 10.1016/S0014-5793(98)01739-6 10025948

[koad211-B39] Weng XY , Wang L, Wang J, Hu Y, Du H, Xu CG, Xing YZ, Li XH, Xiao JH, Zhang QF. Grain number, plant height, and heading date7 is a central regulator of growth, development, and stress response. Plant Physiol. 2014:164(2):735–747. 10.1104/pp.113.231308 24390391 PMC3912102

[koad211-B40] Xiang Y , Tang N, Du H, Ye HY, Xiong LZ. Characterization of OsbZIP23 as a key player of the basic leucine zipper transcription factor family for conferring abscisic acid sensitivity and salinity and drought tolerance in rice. Plant Physiol. 2008:148(4):1938–1952. 10.1104/pp.108.128199 18931143 PMC2593664

[koad211-B41] Yan SJ , Liu Q, Naake T, Huang WJ, Chen MY, Kong Q, Zhang S, Li WY, Li X, Liu QJ, et al *OsGF14b* modulates defense signaling pathways in rice panicle blast response. Crop J. 2021:9(4):725–738. 10.1016/j.cj.2020.10.007

[koad211-B42] Yang C , Ma YM, He Y, Tian ZH, Li JX. OsOFP19 modulates plant architecture by integrating the cell division pattern and brassinosteroid signaling. Plant J. 2018:93(3):489–501. 10.1111/tpj.13793 29205590

[koad211-B43] Yang C , Shen WJ, Yang LM, Sun Y, Li XB, Lai MY, Wei J, Wang CJ, Xu YC, Li FQ, et al HY5-HDA9 module transcriptionally regulates plant autophagy in response to light-to-dark conversion and nitrogen starvation. Mol Plant. 2020:13(3):515–531. 10.1016/j.molp.2020.02.011 32087368

[koad211-B44] Yoshida T , Fujita Y, Sayama H, Kidokoro S, Maruyama K, Mizoi J, Shinozaki K, Yamaguchi-Shinozaki K. AREB1, AREB2, and ABF3 are master transcription factors that cooperatively regulate ABRE dependent ABA signaling involved in drought stress tolerance and require ABA for full activation. Plant J. 2010:61(4):672–685. 10.1111/j.1365-313X.2009.04092.x 19947981

[koad211-B45] Zhao X , Li F, Li K. The 14-3-3 proteins: regulators of plant metabolism and stress responses. Plant Biol (Stuttg). 2021:23(4):531–539. 10.1111/plb.13268 33811408

[koad211-B46] Zhu JK . Salt and drought stress signal transduction in plants. Annu Rev Plant Biol 2002:53(1):247–273. 10.1146/annurev.arplant.53.091401.143329 12221975 PMC3128348

[koad211-B47] Zhu JK . Abiotic stress signaling and responses in plants. Cell 2016:167(2):313–324. 10.1016/j.cell.2016.08.029 27716505 PMC5104190

[koad211-B48] Zong W , Tang N, Yang J, Peng L, Ma SQ, Xu Y, Li GL, Xiong LZ. Feedback regulation of aba signaling and biosynthesis by a bZIP transcription factor targets drought-resistance-related genes. Plant Physiol. 2016:171(4):2810–2825. 10.1104/pp.16.00469 27325665 PMC4972276

