# Supplementary material for: The 14-3-3 protein OsGF14f interacts with OsbZIP23 and enhances its activity to confer osmotic stress tolerance in rice
Source: Plant Cell. 2023 Jul 28;35(11):4173–89. doi: 10.1093/plcell/koad211 (PMC10615203; doi:10.1093/plcell/koad211)
Supplement: koad211_Supplementary_Data [file koad211_supplementary_data.zip › tpc.22.01233Supplemental figures.pdf]

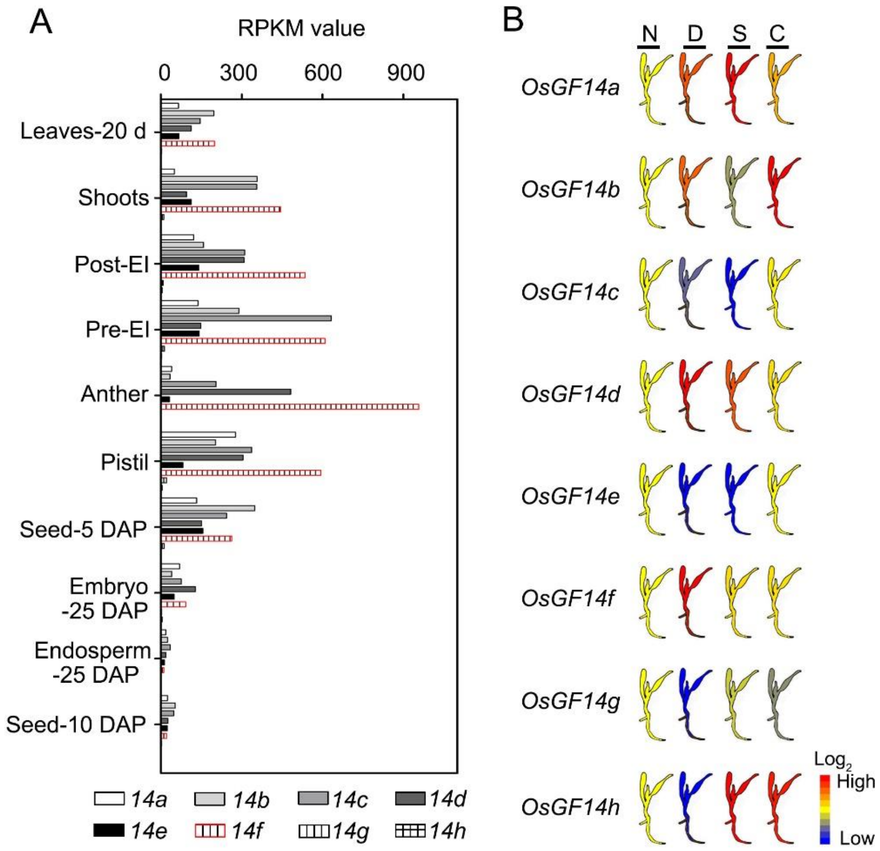

Supplemental Figure S1. Expression analysis of rice 14-3-3 family genes. (Supports Figure 1)

(A) Expression levels of rice 14-3-3 family genes in different tissues at different growth stages. Data was obtained from the RGAP database (http://rice.uga.edu/). Post-EI, Post-emergence inflorescence. Pre-EI, Pre-emergence inflorescence. DAP, Days after pollination.

(B) Expression maps of rice 14-3-3 family genes in response to stress treatments. Data was obtained from the Bio-Analytic Resource for Plant Biology (http://bar.utoronto.ca/). N, normal. D, drought. S, salt. C, cold.

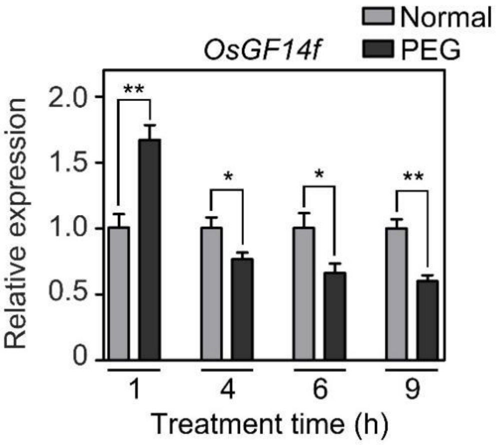

Supplemental Figure S2. The response of *OsGF14f* transcripts to osmotic stress treatment in roots. (Supports Figure 1)

RT-qPCR (reverse transcription quantitative PCR) analysis showing *OsGF14f* gene expression in root following a 20% PEG6000 treatment. Two-week-old Nipponbare seedlings were exposed to a 20% PEG6000 treatment and the roots were harvested at the indicated time points for total RNA extraction and gene expression analysis. Each sample contained five uniform seedlings. Data represents means ± SD of three independent experiments. *P < 0.05, **P < 0.01 (unpaired two-tailed Student's t-test).

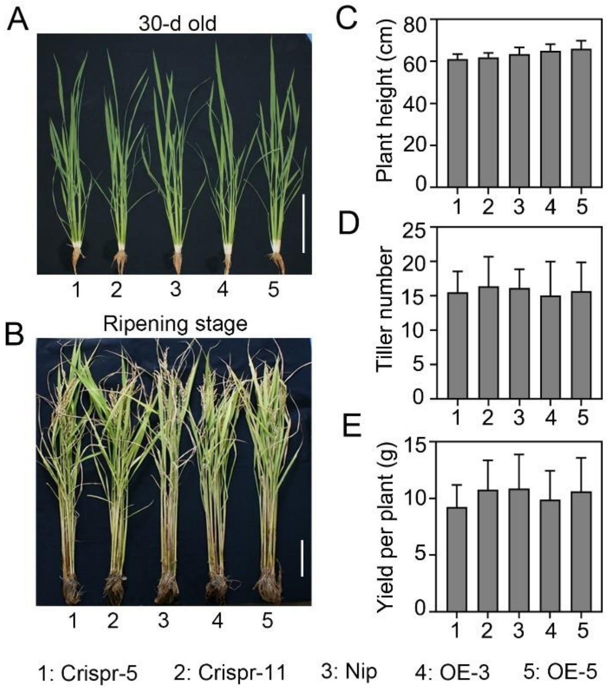

Supplemental Figure S3. Phenotypic observation of *OsGF14f* transgenic plants and Nipponbare under normal growth conditions. (Supports Figure 2)

(A) Phenotypes of 30-d-old *OsGF14f* transgenic and Nipponbare (Nip) seedlings. Scale bars = 10 cm.

(B) Phenotypes of *OsGF14f* transgenic and Nipponbare plants at the ripening stage. Scale bars = 10 cm.

(C-E) Data for plant height (C), tiller numbers (D), and yield per plant (E) of *OsGF14f* transgenic and Nipponbare plants. Data represents means ± SD of sixteen individual plants per genotype. OE, over-expression transgenic line. Crispr, knockout transgenic line by CRISPR/Cas9 gene editing system.

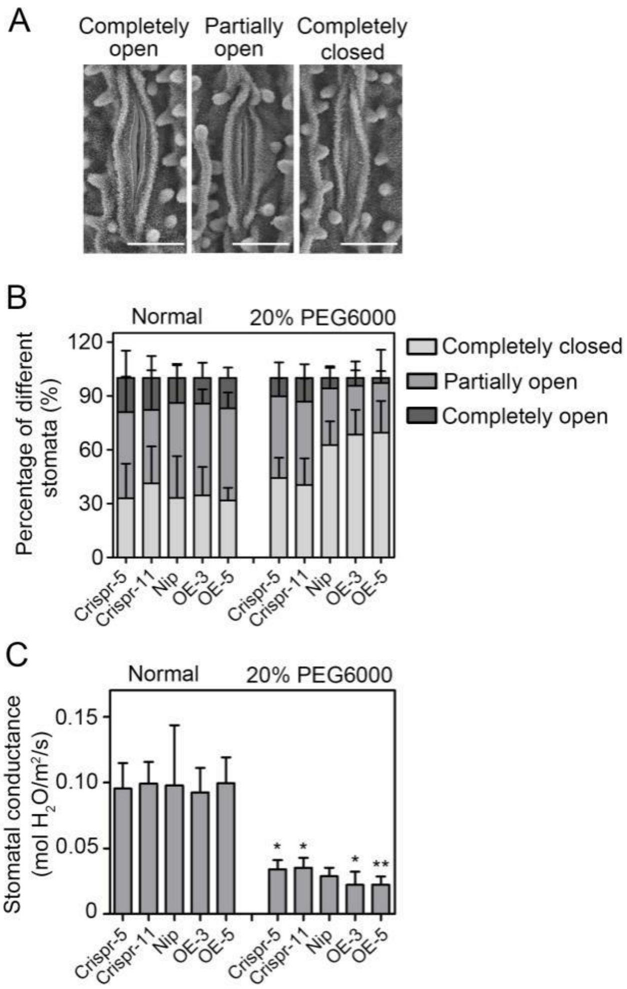

Supplemental Figure S4. The regulation of *OsGF14f* on stomatal aperture and conductance under normal and osmotic stress conditions. (Supports Figure 2)

(A) Scanning electron microscopy images of three levels of stomatal apertures. Scale bars = 10 μm.

(B) The percentage of three levels of stomatal apertures in the leaves of *OsGF14f* transgenic and Nip plants under normal and 20% PEG6000 stress conditions. Data represents means ± SD. (Normal, *n* = 101 stomata for Crispr-5; *n* = 83 stomata for Crispr-11; *n* = 87 stomata for Nip; *n* = 93 stomata for OE-3; *n* = 119 stomata for OE-5; 20% PEG6000, *n* = 78 stomata for Crispr-5; *n* = 104 stomata for Crispr-11; *n* = 100 stomata for Nip; *n* = 87 stomata for OE-3; *n* = 92 stomata for OE-5).

(C) Stomatal conductance of *OsGF14f* transgenic and Nip plants. Data represents means ± SD of sixteen individual plants per genotype. **P < 0.05, **P < 0.01 (unpaired two-tailed Student’s t-test). OE, over-expression transgenic line. Crispr, knockout transgenic line by CRISPR/Cas9 gene editing system.

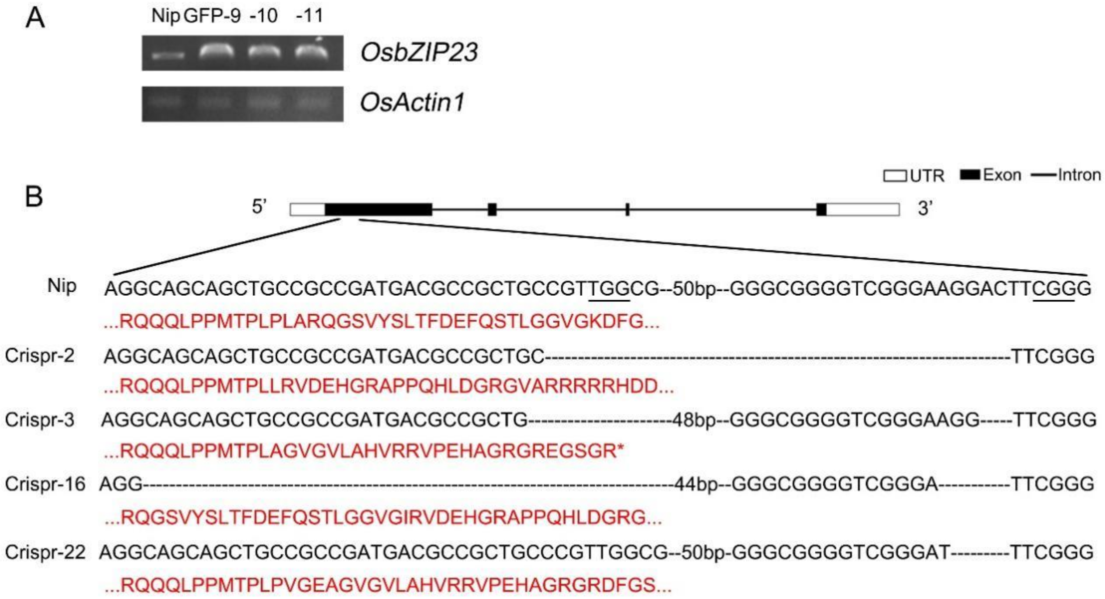

Supplemental Figure S5. Molecular characterization of *OsbZIP23* transgenic plants. (Supports Figures 6-8)

(A) RT-PCR (reverse transcription PCR) analysis of *OsbZIP23* expression levels in Nipponbare (Nip) and *OsbZIP23-GFP* transgenic lines. *OsActin1* was used as a control.

(B) CRISPR/Cas9-mediated mutation of *OsbZIP23*. Protospacer adjacent motifs are underlined. Deletions are shown as dashed lines. Red letters represent amino acid sequences. Asterisks indicate the termination of protein translation. UTR, Untranslated regions.

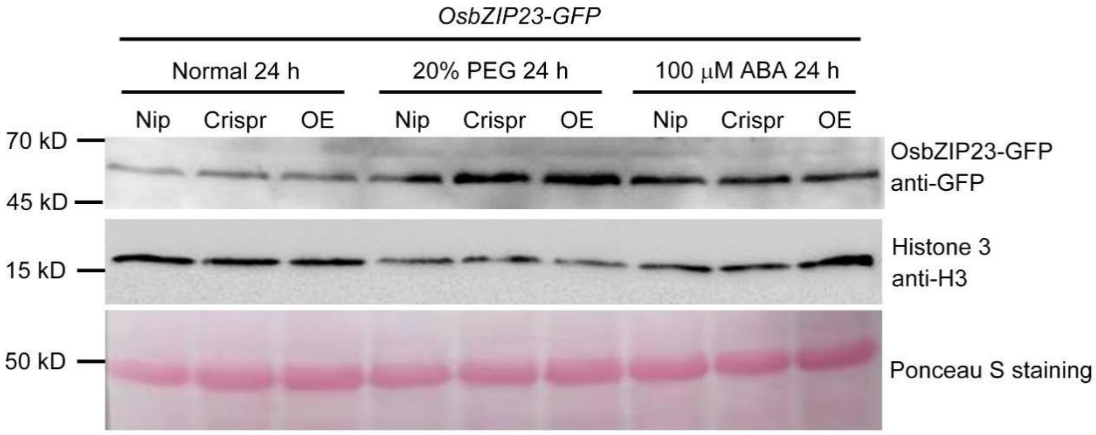

Supplemental Figure S6. OsGF14f does not affect the protein stability of OsbZIP23. (Supports Figure 5)

Seedlings from transgenic lines were grown for 2 weeks and treated with 100 μM ABA (abscisic acid), 20% PEG6000, or a control of 1/2 Kimura B nutrient solution for 24 hours. Total proteins were extracted and subjected to immunoblot analysis using anti-GFP and anti-H3 antibodies. The uniformity of protein loading was verified by ponceau S staining. OE, over-expression transgenic line. Crispr, knockout transgenic line by CRISPR/Cas9 gene editing system.

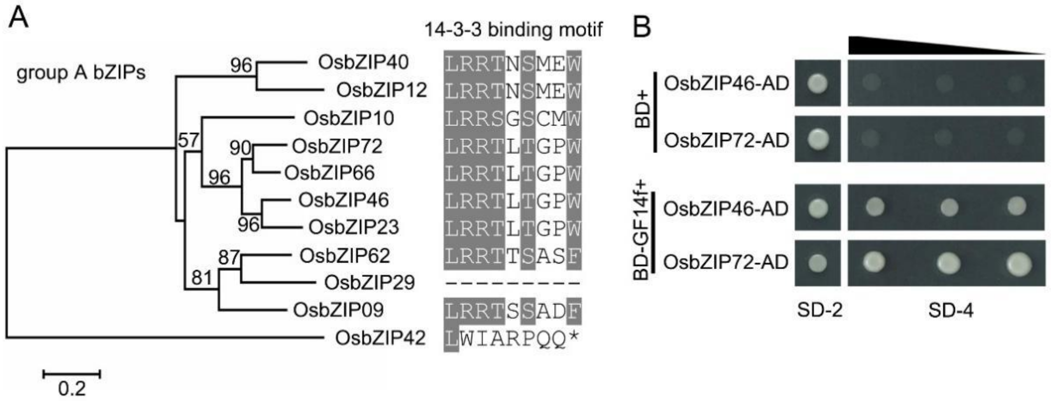

Supplemental Figure S7. OsGF14f interacts with OsbZIP46 and OsbZIP72. (Supports Figure 7)

(A) Phylogenetic relationship of rice group A bZIPs. The phylogenetic tree was constructed based on the sequence alignments of the OsbZIP proteins using Mega 7.0 by the neighbor-joining method. Bootstrap testing was conducted with 1000 replicates. Values are indicated at the nodes of branches. The scale bar indicates the average number of amino acid substitutions per site. Conserved amino acids are shown on a gray background.

(B) Yeast two-hybrid assays showing the interaction between OsGF14f with OsbZIP46 and OsbZIP72. Protein interaction was determined by the growth of the yeast cells co-transformed with various combinations of the plasmids on both a synthetic dropout medium lacking Leu and Trp (SD-2) and a synthetic dropout medium lacking Leu, Trp, His, and adenine (SD-4). AD, pGADT7. BD, pGBKT7. Black triangles represent the concentration gradients.
