# Supplementary material for: The 14-3-3 protein OsGF14f interacts with OsbZIP23 and enhances its activity to confer osmotic stress tolerance in rice
Source: Plant Cell. 2023 Jul 28;35(11):4173–89. doi: 10.1093/plcell/koad211 (PMC10615203; doi:10.1093/plcell/koad211)
Supplement: koad211_Supplementary_Data [file koad211_supplementary_data.zip › tpc.22.01233Supplemental File S1.pdf]

Supplemental File S1. Genetics module.
Details related to the characterization of mutant or transgenic lines.

Mutant or transgenic line	Description
OsGF14f-Crispr	Knockout plants of OsGF14f were obtained through CRISPR/cas9 method, and the mutation information was shown in Figure 2D.
OsGF14f-OE	Overexpression lines in Nipponbare (Nip) background.
OsbZIP23-Crispr	Knockout plants of OsbZIP23 were obtained through CRISPR/cas9 method, and the mutation information was shown in Supplementary Figure 5B.
OsbZIP23-GFP	Overexpression lines in Nipponbare (Nip) background.
OsbZIP23-GFP-9/*OsGF14f*-Crispr-5	*OsbZIP23-GFP*-9 and *OsGF14f*-Crispr-5 were crossed to generate *OsbZIP23-GFP*-9/*OsGF14f*-Crispr-5.<br>Homozygote plants were used for analysis.
OsGF14f-OE-5/ *OsbZIP23*-Crispr-3	*OsGF14f*-OE-5 and *OsbZIP23*-Crispr-3 were crossed to generate *OsGF14f*-OE-5/ *OsbZIP23*-Crispr-3.<br>Homozygote plants were used for analysis.
