# Supplementary figures and images for: The 14-3-3 protein OsGF14f interacts with OsbZIP23 and enhances its activity to confer osmotic stress tolerance in rice
Source: Plant Cell. 2023 Jul 28;35(11):4173–89. doi: 10.1093/plcell/koad211 (PMC10615203; doi:10.1093/plcell/koad211)

Supplemental File S2. Original blot images for Figure 4F.

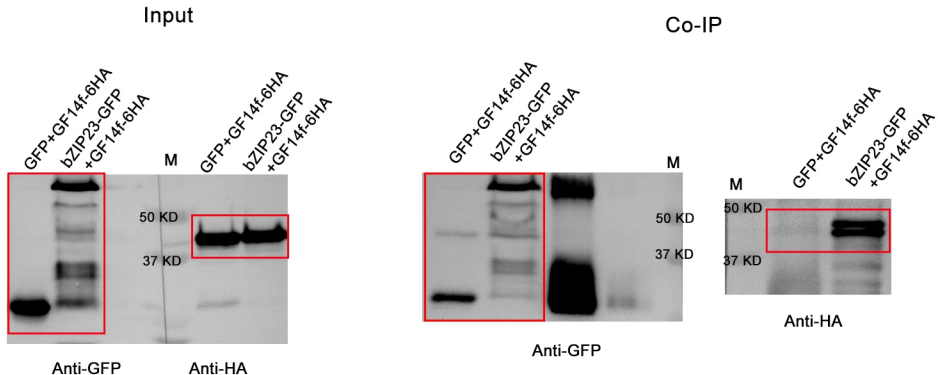

Supplement: koad211_Supplementary_Data [file koad211_supplementary_data.zip › tpc.22.01233Supplemental File S2.pdf]
